# A rasterized building footprint dataset for the United States

**DOI:** 10.1038/s41597-020-0542-3

**Published:** 2020-06-29

**Authors:** Mehdi P. Heris, Nathan Leon Foks, Kenneth J. Bagstad, Austin Troy, Zachary H. Ancona

**Affiliations:** 1 College of Architecture and Planning, University of Colorado Denver, University of Colorado Denver, Denver, CO 80202 USA; 2 Apogee Engineering LLC, contracted to U.S. Geological Survey, 8610 Explorer Dr #305, Colorado Springs, CO 80920 USA; 3 Geosciences & Environmental Change Science Center, U.S. Geological Survey, Denver, CO 80225 USA

**Keywords:** Geography, Environmental sciences, Environmental social sciences, Environmental impact

## Abstract

Microsoft released a U.S.-wide vector building dataset in 2018. Although the vector building layers provide relatively accurate geometries, their use in large-extent geospatial analysis comes at a high computational cost. We used High-Performance Computing (HPC) to develop an algorithm that calculates six summary values for each cell in a raster representation of each U.S. state, excluding Alaska and Hawaii: (1) total footprint coverage, (2) number of unique buildings intersecting each cell, (3) number of building centroids falling inside each cell, and area of the (4) average, (5) smallest, and (6) largest area of buildings that intersect each cell. These values are represented as raster layers with 30 m cell size covering the 48 conterminous states. We also identify errors in the original building dataset. We evaluate precision and recall in the data for three large U.S. urban areas. Precision is high and comparable to results reported by Microsoft while recall is high for buildings with footprints larger than 200 m2 but lower for progressively smaller buildings.

## Abstract (summary)

**Table:** 

Measurement(s)	building • building footprint • area • building count
Technology Type(s)	computational modeling technique
Sample Characteristic - Environment	city
Sample Characteristic - Location	contiguous United States of America

Machine-accessible metadata file describing the reported data: 10.6084/m9.figshare.12444776

## Background & Summary

Building footprints are a critical environmental descriptor. Microsoft produced a U.S.-wide vector building dataset in 2018^1^ that was generated from aerial images available to Bing Maps using deep learning methods for object classification^2^. The main goal of this product has been to increase the coverage of building footprints available for OpenStreetMap. Microsoft identified building footprints in two phases; first, using semantic segmentation to identify building pixels from aerial imagery using Deep Neural Networks and second, converting building pixel blobs into polygons. The final dataset includes 125,192,184 building footprint polygon geometries in GeoJSON vector format, covering all 50 U.S. States, with data for each state distributed separately. These data have 99.3% precision and 93.5% pixel recall accuracy^2^. Temporal resolution of the data (i.e., years of the aerial imagery used to derive the data) are not provided by Microsoft in the metadata.

Using vector layers for large-extent (i.e., national or state-level) spatial analysis and modelling (e.g., mapping the Wildland-Urban Interface, flood and coastal hazards, or large-extent urban typology modelling) is challenging in practice. Although vector data provide accurate geometries, incorporating them in large-extent spatial analysis comes at a high computational cost. We used High Performance Computing (HPC) to develop an algorithm that calculates six summary statistics (described below) for buildings at 30-m cell size in the 48 conterminous U.S. states, to better support national-scale and multi-state modelling that requires building footprint data. To develop these six derived products from the Microsoft buildings dataset, we created an algorithm that took every single building and built a small meshgrid (a 2D array) for the bounding box of the building and calculated unique values for each cell of the meshgrid. This grid structure is aligned with National Land Cover Database (NLCD) products (projected using Albers Equal Area Conic system), enabling researchers to combine or compare our products with standard national-scale datasets such as land cover, tree canopy cover, and urban imperviousness^3^.

Locations, shapes, and distribution patterns of structures in urban and rural areas are the subject of many studies. Buildings represent the density of built up areas as an indicator of urban morphology or spatial structures of cities and metropolitan areas^4,5^. In local studies, the use of vector data types is easier^6,7^. However, in regional and national studies a raster dataset would be more preferable. For example in measuring the spatial structure of metropolitan areas a rasterized building layer would be more useful than the original vector datasets^8^.

**Fig. 1:**
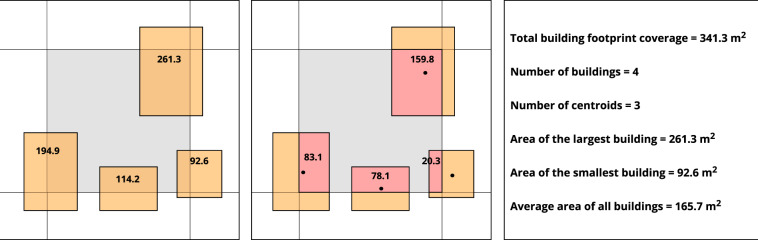
Example cell and intersecting buildings to illustrate how summary values are calculated. Left: The total area of each building is shown. Center: Only the building area within the highlighted cell is shown.

Our output raster products are: (1) total building footprint coverage per cell (m^2^ of building footprint per 900 m^2^ cell); (2) number of buildings that intersect each cell; (3) number of building centroids falling within each cell; (4) area of the largest building intersecting each cell (m^2^); (5) area of the smallest building intersecting each cell (m^2^); and (6) average area of all buildings intersecting each cell (m^2^). The last three area metrics include building area that falls outside the cell but where part of the building intersects the cell (Fig. 1). These values can be used to describe the intensity and typology of the built environment.

Since the data^9^ and software^10^ from this project are publicly available, the code could be used to generate similar raster summary layers in other areas where building data are available. For example, Microsoft has published similar building footprint datasets for Canada^11^ and Eastern Africa^12^, and similar data might be produced in other locations or for different time periods.

## Methods

To create a rasterized summary building footprint dataset, we first selected an appropriate spatial resolution. We assumed that most potential U.S.-based applications of such data would also use data from NLCD and other Landsat products, which have 30-m resolution. Aligning the rasterized building footprint summary with NLCD products would provide the opportunity for future analysts to use building data along with land cover, tree canopy cover, and impervious surface cover data. Although 30-m resolution may be an appropriate scale for the U.S., our code is flexible enough to calculate the same values for different spatial resolutions as well.

To carry out the rasterization procedure, we used open-source Python libraries. Box 1 presents pseudo-code illustrating our method to intersect building shapes with the cells of a raster dataset and then compute the summary statistics needed to produce the six building dataset outputs. Our software is also open-source^10^. The Python packages we used include “Fiona”^13^ to read in the building shapes as a list of shape objects; “Shapely”^14^ to perform the intersections between raster cells and building shapes; and “Rasterio”^15^ to obtain raster information and write our final summary layers to GeoTIFFs. All arrays were + using the NumPy package^16^. For each cell, we found the intersection of the 30-m grid cells with each building and calculated the six values in our dataset. Box 1 elaborates the main procedure to calculate these summary values.

Two computational aspects required further consideration when implementing this algorithm at a large scale. First, reading in an entire set of building shapes upon instantiation of the algorithm leads to an unnecessarily large time and memory overhead. Instead, we simply open the shapefile (generated from GeoJSON) with Fiona without reading the entire contents and iteratively process each shape. This approach has the benefit of reducing memory and initial time overhead. Second, to ensure that the alignment of our summary raster layers matches the alignment of each state (U.S. state NLCD grids are seamless), we used Rasterio to read the geometry of each state’s raster grid and used that geometry to initialize our arrays. The state-wide GeoJSON layers do not have duplicated buildings on state boundaries. Therefore, putting together state layers does not cause double counting. For example, if a cell has intersecting buildings with two adjacent states the values of the state-wide layer will reflect the buildings of one state only. Since we only require the geometry of the state raster grid, we do not read its cell values since this would impose a high memory and time overhead.

We implemented this algorithm both in serial and in parallel using mpi4py^17^. The parallel algorithm behaves similarly to the serial algorithm with the exception that polygons in the shapefile are distributed among available Central Processing Unit (CPU) cores. We used the CPU cores in a master-worker paradigm where a single master rank distributes the polygons to each worker in user-specified chunks. This approach was necessitated by the inability to read in all geometry objects of one state at once due to large memory and time requirements. Instead, the master rank opens the shapefile and iteratively reads and distributes chunks of polygons from the shapefile. Once a worker rank finishes processing its chunk of polygons, it requests the next chunk from the master. An unfortunate side effect of this paradigm is the duplication of memory on each CPU core to store the rasterized summary statistics. For very large raster layers, or large states in our dataset, this requires a large amount of RAM, however for small to medium examples a standard laptop or desktop will suffice. To overcome the memory limitations of the largest states, we used the USGS Yeti supercomputer^18^, which enabled access to a large shared-memory machine. We also tried an out-of-memory approach HDF5 with parallel file access, however the increase in computation time due to the atomic operations of our statistics was prohibitive.

Box 1 Intersection of building shapes with a raster layer:

1. **shps ←** Read in the building shapefile
2. **geom**= {x0, y0, dx, dy, nx, ny} ← Origin, cell size, and number of cells in the raster
3. **count** = 0 ← Initialize an integer array with shape(nx, ny)
4. **area** = 0.0 ← Initialize a float array with shape(nx, ny)
5. **intersectArea** = 0.0 ← Initialize a float array with shape(nx, ny)
6. **min** = huge (a large number) ← Initialize a float array with shape(nx, ny)
7. **max** = 0.0 ← Initialize a float array with shape(nx, ny)
8. **centroidCount** = 0 ← Initialize an integer array with shape(nx, ny)
9. **for** each *shp* in **shps do**
10. **box** ← Bounding box for current *shp*
11. **for** each *cell* in **box ∩** *shp* **do**
12. **count**(*cell*) += 1
13. **area**(*cell*) += *shp*.area
14. **intersectArea**(cell) += *shp*.intersect(*cell*).area
15. **min**(*cell*) = minimum(min(*cell*), *shp*.area)
16. **max**(*cell*) = maximum(max(*cell*), *shp*.area)
17. **if** *shp*.centroid ? *cell* **do**
18. **centroidCount**(*cell*) += 1
19. **where** count != 0
20. average = area / count
21. Write each summary layer to GeoTiff

## Data Records

**Fig. 2:**
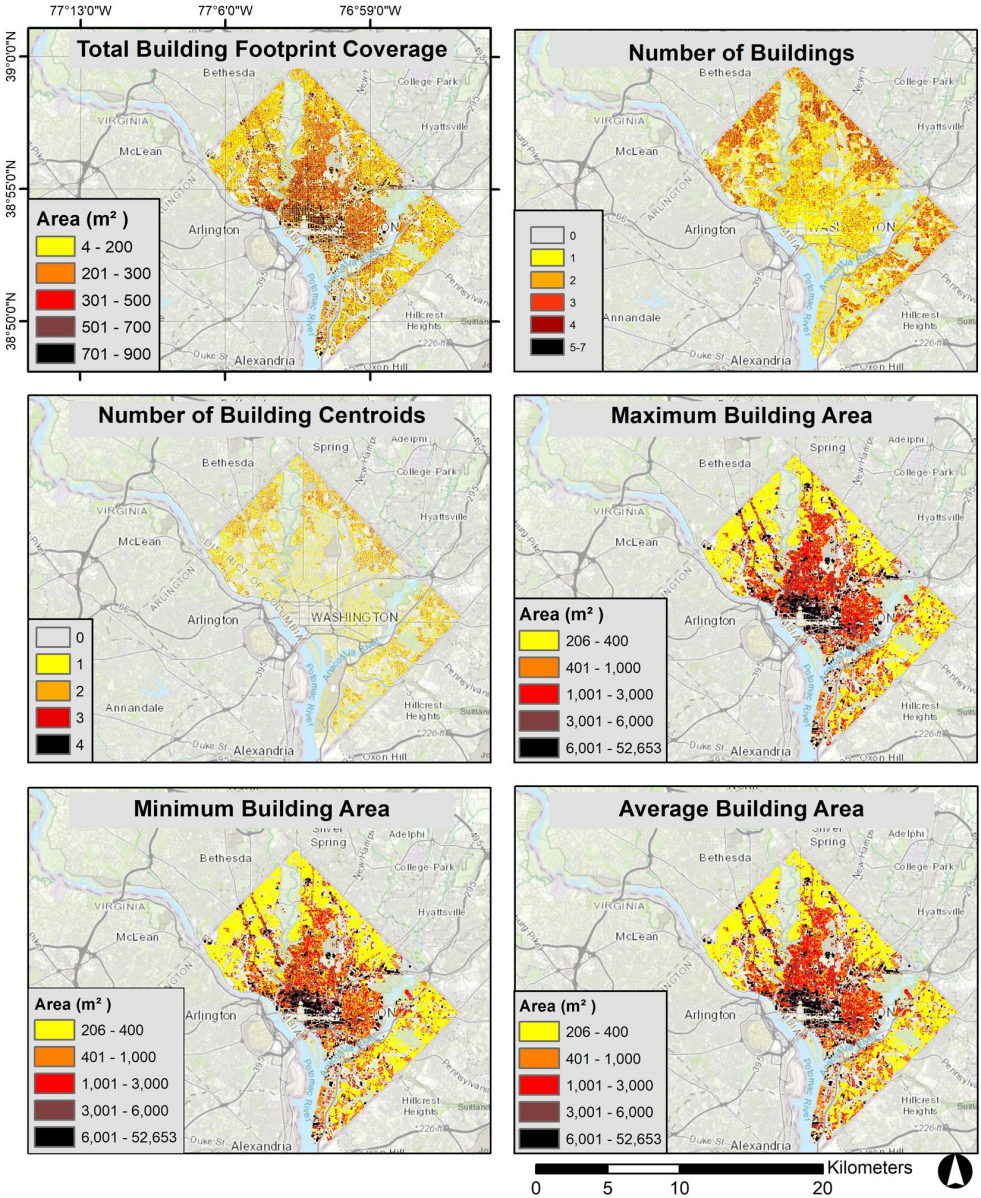
Rasterized summary layers of building footprints for Washington, DC.

The final data product has six raster layers for all U.S. states and the District of Columbia except Alaska and Hawaii. Figure 1 shows an example cell intersecting with four buildings. In this case, we first count the number of intersecting buildings (four) and building centroids (three) in each grid cell, then we calculate the total area of the cell that is covered by building footprints (346 m^2^), in addition to the area of the average, smallest, and largest intersecting buildings (165.7 m^2^, 92.6 m^2^, and 261.3 m^2^, respectively). Figure 2 shows the rasterized summary layers of building footprints for the District of Columbia.

The data are available from the U.S. Geological Survey’s ScienceBase Catalog^9^. The publicly available data include two categories: (1) state-level layers (49 zipped folders) and (2) conterminous U.S. layers. A metadata XML file is included in the data package. In the state-wide category, each zipped folder has six summary rasters. The raster layers represent (1) [stateName]_sum: total footprint coverage; (2) [stateName]_cnt: number of unique buildings intersecting each cell; (3) [stateName]_centroids: number of building centroids falling inside each cell; and area of the (4) [stateName]_avg: average; (5) [stateName]_min: smallest, and (6) [stateName]_max: largest area of buildings that intersect each cell. In the conterminous U.S. the same six layers are included.

## Technical Validation

The rasterized building footprint output dataset represents the vector building footprints data with six raster layers. The rasterized data do not degrade or improve the accuracy of the vector layers. Microsoft has validated the vector building dataset and reported 99.3% precision and 93.5% recall as building matching metrics^2^. In the Machine Learning literature, precision refers to the ratio of relevant instances (buildings) among the retrieved instances. Recall is the fraction of the total amount of relevant instances that were actually retrieved^19^. To calculate recall and precision we identified two types of error in the Microsoft dataset: (1) undercounting existing buildings (the non-detection of building objects) and (2) overcounting buildings (identifying non-building objects or patterns as buildings). To explore the vector building footprint’s accuracy, we used high-resolution building datasets for three cities/regions: (1) city of Denver, CO, (2) New York, NY, and (3) Los Angeles County, CA. Building footprint datasets of Denver^20^ and New York^21^ are produced from orthoimagery captured in 2014 and 2015, respectively. The Los Angeles County building dataset^22^ is produced from stereo imagery captured in 2008. Since the Los Angeles County data is relatively older than Denver and New York, we expect to observe a slightly lower precision rate due to the absence of buildings that have been constructed since 2008.

### Undercounting

**Fig. 3:**
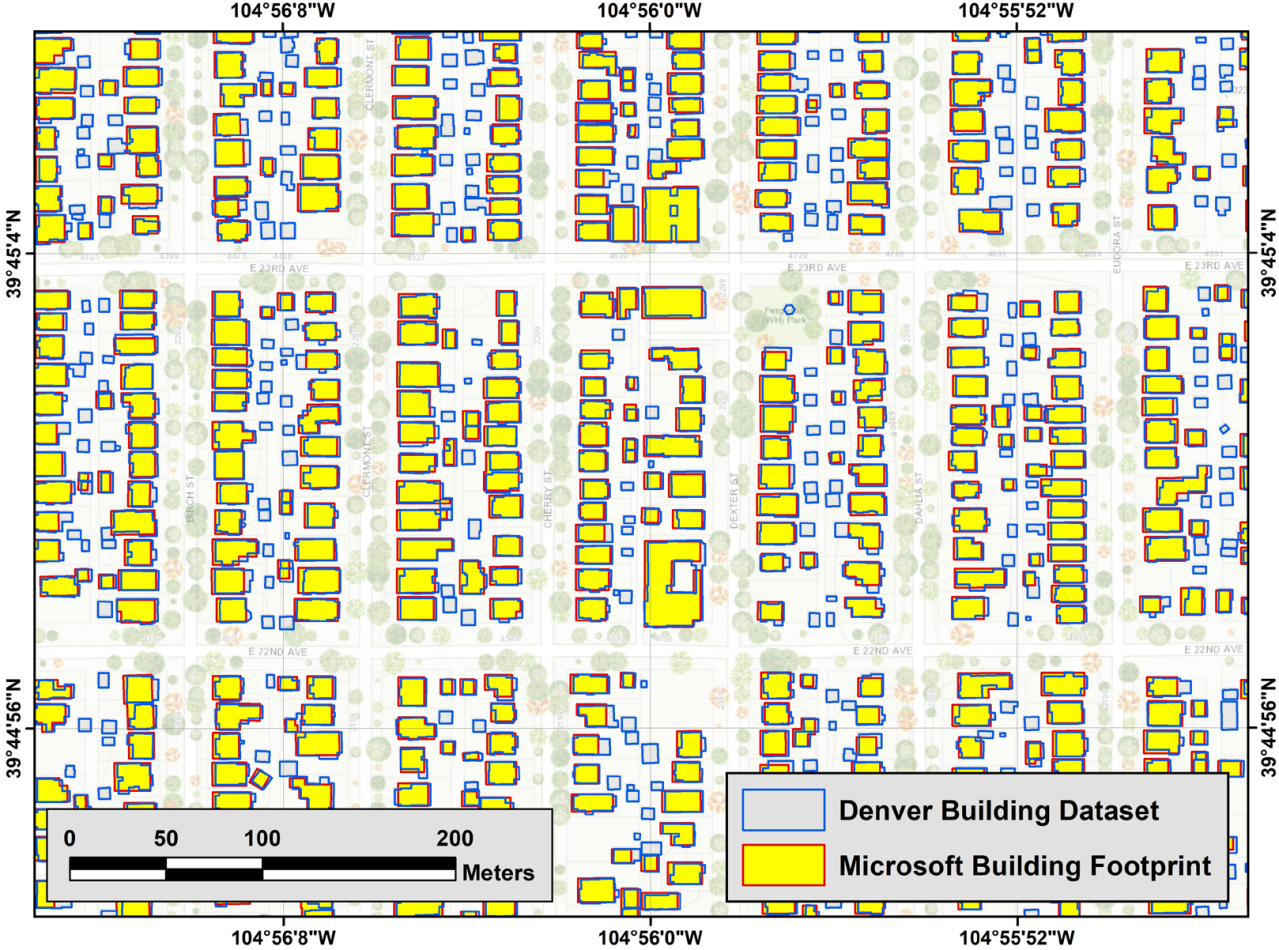
Neighborhood-scale comparison of City of Denver and Microsoft building footprint datasets.

**Fig. 4:**
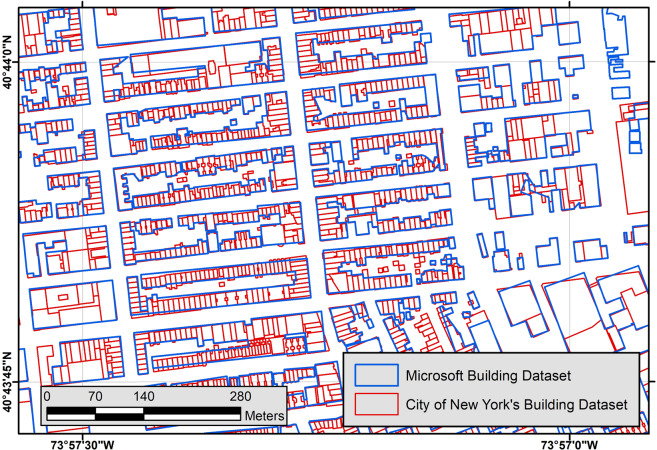
Histogram of the area of missing buildings in the Microsoft dataset for Denver, CO, New York, NY, and Los Angeles County, CA.

**Fig. 5:**
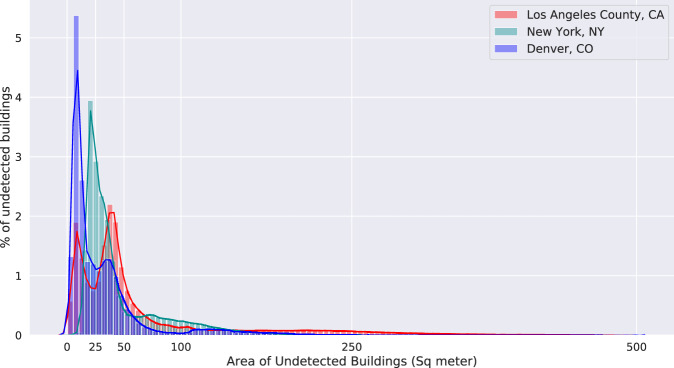
Missing tiles in San Jose, CA. Existing Microsoft data are overlaid on the aerial image to show data gaps.

Our evaluation shows that Microsoft’s algorithm fails to detect small buildings (generally those smaller than 100 m^2^) in all three regions. Most of the undetected buildings are accessory units such as garden sheds or detached garages in this example area of Denver (Fig. 3). Figure 4 shows the histogram of the size of undetected buildings in Denver, Los Angeles, and New York City and highlights the large number of small undetected buildings. We also identified systematic gaps in the Microsoft data for some geographic areas. These larger gaps seem to have a tile pattern, where aerial photos may have been unavailable to the Microsoft building detection algorithm, for example in Austin, TX and San Jose, CA (Fig. 5). In Denver, a relatively small tile gap resulted in 7,478 missing buildings (2.6% of the city’s total).

**Fig. 6:**
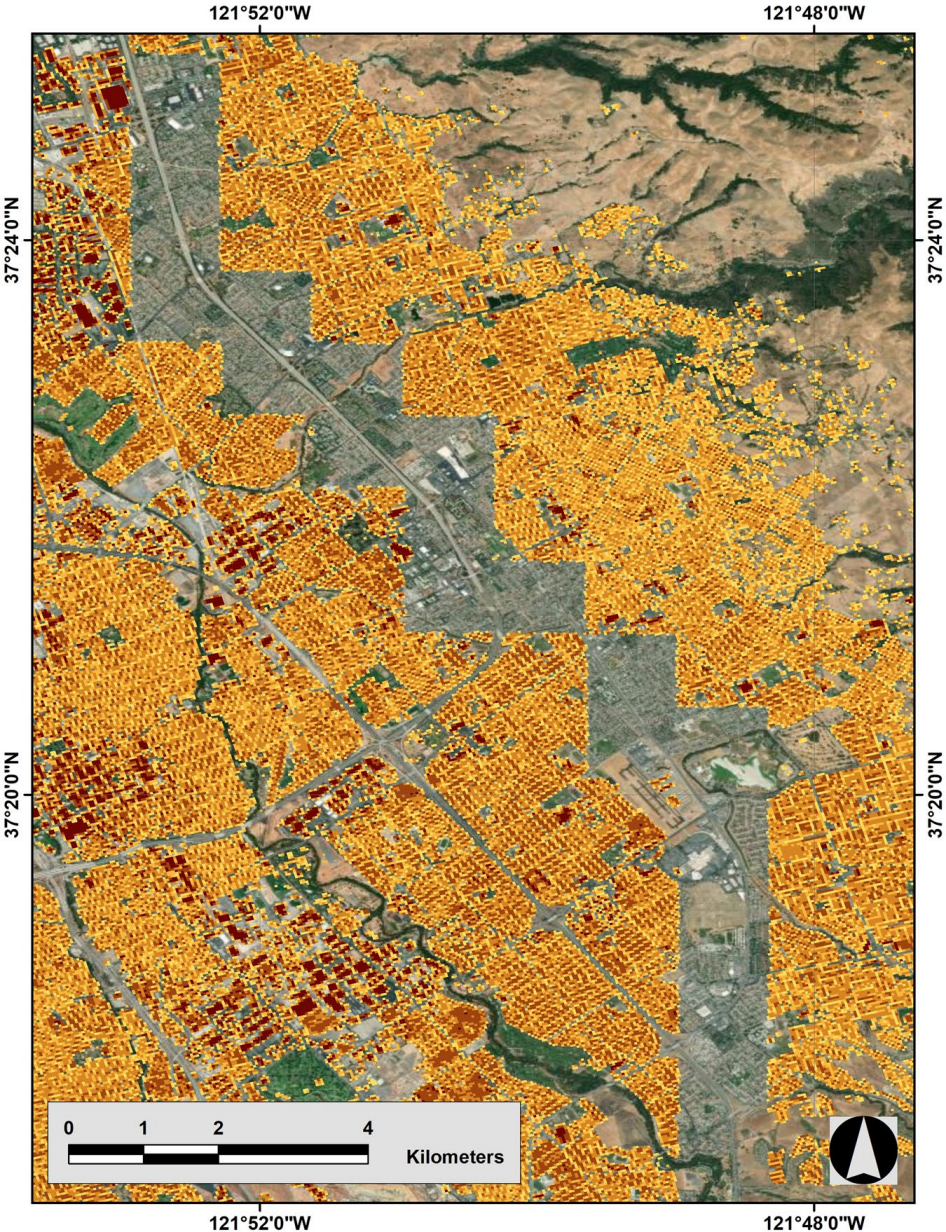
Consolidated buildings in Manhattan, New York, NY.

In New York City, we also noticed that attached buildings with similar heights are consolidated to form a large block of single polygons (Fig. 6). Although these polygons do not substantially affect the total area of footprint coverage, they affect counts, counts of centroids, and average area of buildings. This error must be considered in high-density areas with where attached buildings are common.

### Overcounting

**Fig. 7:**
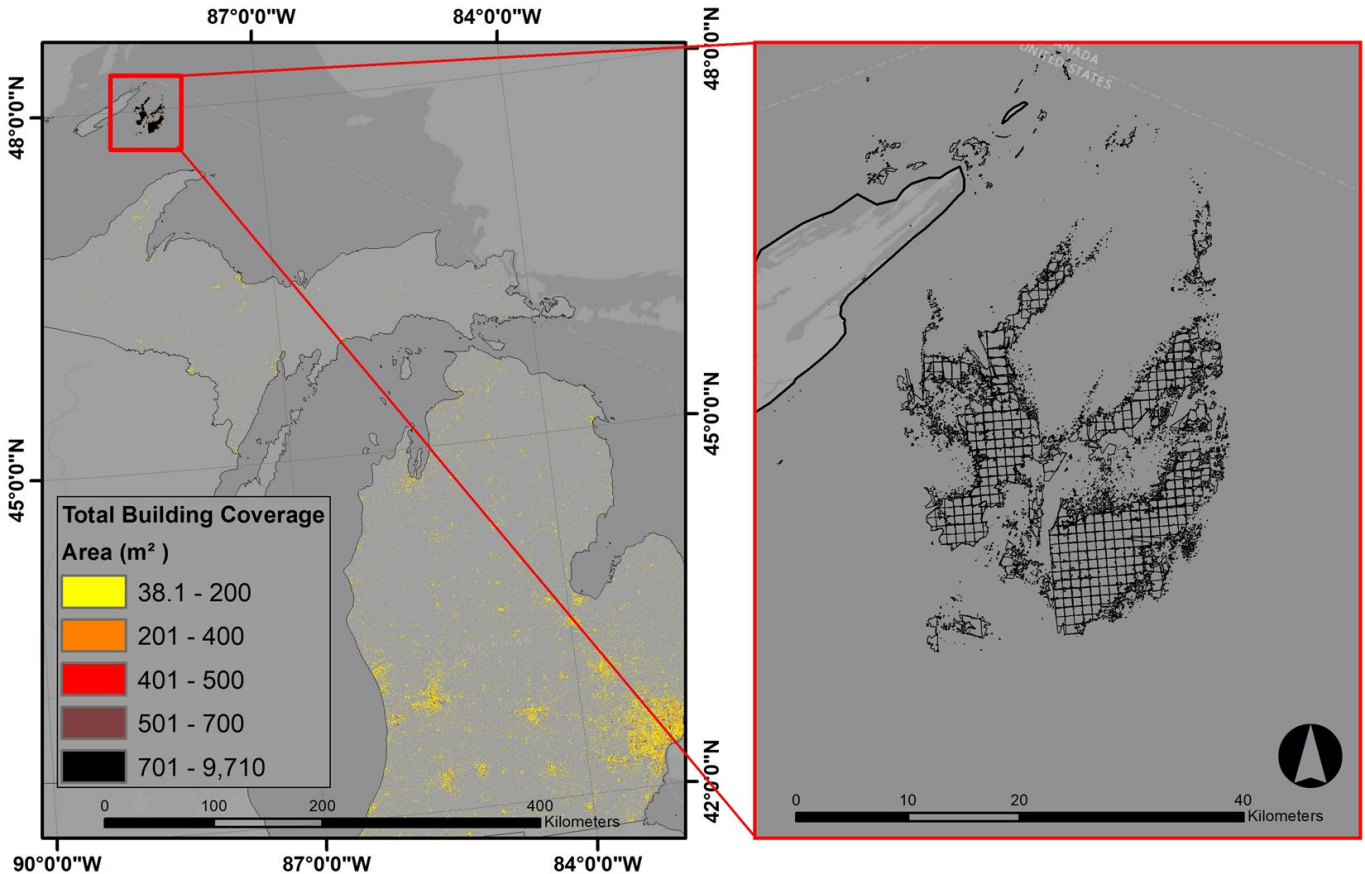
Data artifacts in Lake Superior (Left: the location of data artifacts; Right: overlapping building polygons in the Lake).

In some cases, the Microsoft building dataset has identified objects and patterns as buildings where there is no structure. These data artifacts are rare but can be problematic when the layer is being used in a national study. These polygons are most likely detected based on the reflectance of objects that create a building-like pattern. We identified these artifacts in lakes, rivers, and snow-covered areas at high altitude. Figure 7 illustrates a cluster of polygons in Lake Superior where there clearly is no structure. This misidentification is probably due to reflectance patterns in the aerial images. Another problem in the Microsoft dataset occurs when overlapping polygons generate values larger than 900 m^2^ for the total footprint coverage value within a grid cell. We describe these errors and data artifacts in the Supplementary Information.

#### Precision and recall

**Fig. 8:**
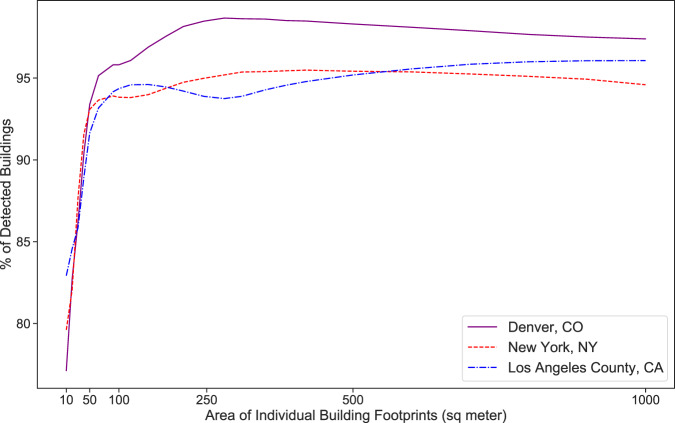
Precision accuracy metric for Microsoft building footprint dataset.

To report the precision metric, we calculated the ratio of accurately identified building objects to all detected objects in the Microsoft dataset. Precision of the Microsoft dataset is 99.5%, 99.1%, and 98.2% in Denver, New York, and Los Angeles County, respectively (Table 1). We also calculated precision for different area ranges. For buildings larger than 100 m^2^ precision is above 99% for Denver and New York and is 98.8% for Los Angeles County (Fig. 8). This is consistent Microsoft’s reported precision. The lower precision of Los Angeles County can be attributed to the relatively older building data that we used as for ground truthing.

**Table 1:** Accuracy assessment of Microsoft building footprint dataset in three U.S. cities.

	Precision			Recall		
	Los Angeles County, CA	NYC, NY	Denver, CO	Los Angeles County, CA	NYC, NY	Denver, CO
All area ranges	98.20%	99.10%	99.50%	73.00%	36.50%	63.80%
10 m^2^ < = area <50 m^2^	94.50%	93.30%	97.80%	28.90%	6.50%	12.80%
50 m^2^ < = area <100 m^2^	96.90%	98.90%	99.20%	57.20%	19.20%	63.80%
100 m^2^ < = area <200 m^2^	98.80%	99.50%	99.70%	86.90%	55.20%	86.90%
200 m^2^ < = area <500 m^2^	98.90%	99.20%	99.50%	92.80%	99.70%	99.60%
500 m^2^ < = area <1500 m^2^	97.50%	99.40%	99.70%	94.70%	98.00%	93.70%

**Fig. 9:**
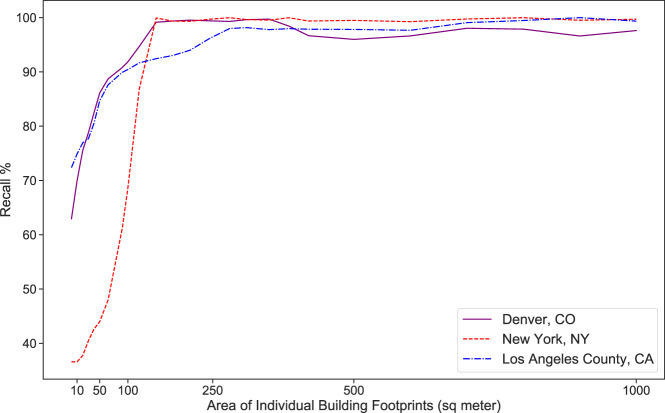
Recall accuracy metric for Microsoft building footprint dataset.

To calculate recall, we subtracted falsely detected objects from all detected objects by Microsoft data and divided by all instances (using the high-resolution data). As expected, recall values are fairly low in area ranges smaller than 150 m^2^. When all area ranges are assessed, the overall recall ratios are 63.8%, 36.5%, and 73.0% for Denver, New York, and Los Angeles County respectively. Recall values for buildings larger than 200 m^2^ are about 99% for Denver and New York and 93% for Los Angeles County (Fig. 9). Our validation shows that studies that use this building dataset need to note the purpose that this data serves. If small buildings do not matter significantly, then this dataset has an acceptable accuracy. Additionally, we identified a series of common issues in this dataset that are documented in the Supplementary Information.

## Usage Notes

A rasterized building footprint dataset can be helpful in a wide range of studies related to built environments^8,23,24^. A particularly relevant use of these data would be to evaluate spatially disaggregated measures of urbanization across the country. Studies that evaluate land cover, land use, and human settlement patterns would benefit from the use of this dataset^25,26^. Most major cities in the U.S. now use high-resolution orthoimagery and LiDAR to produce their own building footprint datasets^27^. Therefore, the Microsoft dataset is particularly helpful where such data are not available.

The rasterized layers match the resolution and cell alignment of NLCD land cover, tree canopy, and impervious cover products. This is an advantage when all or some of these layers are used in models since rescaling, shifting, or retiling need not be carried out. The summary layers can also be used independently or in combination. For example, comparing the total building footprint coverage in a cell with the average building area would yield the built-up density in a cell. A different application of our data might focus on the size of buildings to classify use types or residential density. In that case, the average building area might be most useful. When the number of structures is the subject of interest, the building centroid count might be useful in a certain geography.

The original vector dataset provided by Microsoft is appropriate for small-extent applications (e.g., city- or metropolitan-area scale studies). However, in large-extent studies (multistate or national-scale), using the vector data format would require intensive computational power and could require complicated geoprocessing procedures. In such cases, using the rasterized products would be substantially faster. Finally, another advantage of rasterized layers is the capacity to convert them to arrays in programming environments such as Numpy arrays for Python which is computationally attractive for large-extent applications.

## Supplementary information

Supplementary Information

## Data Availability

Our software is available through U.S. Geological Survey code repository (https://doi.org/10.5066/P9XZCPMT)^10^. Our serial code is also available in our Github page: https://github.com/mehdiheris/RasterizingBuildingFootprints.
