# Author Correction: A rasterized building footprint dataset for the United States

**DOI:** 10.1038/s41597-020-00585-0

**Published:** 2020-07-16

**Authors:** Mehdi P. Heris, Nathan Leon Foks, Kenneth J. Bagstad, Austin Troy, Zachary H. Ancona

**Affiliations:** 1 College of Architecture and Planning, University of Colorado Denver, University of Colorado Denver, Denver, CO 80202 USA; 2 Apogee Engineering LLC, contracted to U.S. Geological Survey, 8610 Explorer Dr #305, Colorado Springs, CO 80920 USA; 3 Geosciences & Environmental Change Science Center, U.S. Geological Survey, Denver, CO 80225 USA

Correction to: *Scientific Data*10.1038/s41597-020-0542-3, published online 29 June 2020

The original version of this Data Descriptor incorrectly labelled Figure 4 as Figure 5, Figure 5 as Figure 6 and Figure 6 as Figure 4. The ordering of Figures 4, 5, and 6 has now been corrected in both the HTML and PDF versions of this Data Descriptor.

