# Supplementary material for: A rasterized building footprint dataset for the United States
Source: Sci Data. 2020 Jun 29;7:207. doi: 10.1038/s41597-020-0542-3 (PMC7324622; doi:10.1038/s41597-020-0542-3)
Supplement: Supplementary file 1 — Supplementary Information [file 41597_2020_542_MOESM1_ESM.pdf]

Supplementary Information

Table of Contents:

SI Table 1	Page 2
SI Figure 1	Page 3
SI Figure 2	Page 4
SI Figure 3	Page 5
SI Figure 4	Page 6
SI Figure 5	Page 7
SI Figure 6	Page 8
SI Figure 7	Page 9
SI Figure 8	Page 10
SI Figure 9	Page 11
SI Figure 10	Page 12
SI Figure 11	Page 13
SI Figure 12	Page 14
SI Figure 13	Page 15
SI Figure 14	Page 16
SI Figure 15	Page 17
SI Figure 16	Page 18
SI Figure 17	Page 19
SI Figure 18	Page 20
SI Figure 19	Page 21

The following supplemental figures provide an overview of the common issues found throughout our manual QA/QC process. We found that only 4 states (Maine, Montana, Rhode Island, and Wyoming) and District of Columbia have maximum raster values of 900 m^2; the remaining 43 states all have values above 900 m^2. This confirms that there are overlapping polygons in almost every state, although the severity of their overlap varies.

We found nine common errors in the original Microsoft building shapefiles that users should be aware of. These are errors generated during the creation of the original dataset that are compounded through development of the raster datasets created in this analysis.

The errors found in this process and reported here are corrected in all output layers to improve the accuracy to some extent. However, there are many other instances of error remaining in the dataset. Our report helps future studies to address the error of this data systematically.

SI Table 2 Common types of error in overcounting building footprints

Error Code	Error Description	Example Figures
ERR1: Overlapping Buildings	Overlapping polygons	SI: 11 and 16
ERR2: Open Water	Building polygons are present in open water and rivers	SI: All except 3, 4, 10, 11 and 18
ERR3: High Elevation/Snow Reflectance	Building polygons found at high elevations with snow cover	SI: 3 and 4
ERR4: Salt/White Sand Reflectance	Building polygons found on salt flats or white sand with high reflectance	SI: 11 and 19
ERR5: Trains/Transportation	Building polygons found where trains and other forms of large transportation infrastructure exist	SI: 16
ERR6: Mining/Bare Ground Reflectance	Building polygons found on areas with bare ground, quarrying, and mining	SI: 18
ERR7: Extent Issues/Misclassification	Building polygons found in areas where no buildings exist	SI: 5, 10, 11 and 19
ERR8: Missing tiles	Big systematic gaps are observed in multiple states	Figure 5 of the manuscript
ERR9: Consolidating multiple buildings	Multiple attached buildings were consolidated to form a large block.	Figure 4 of the manuscript

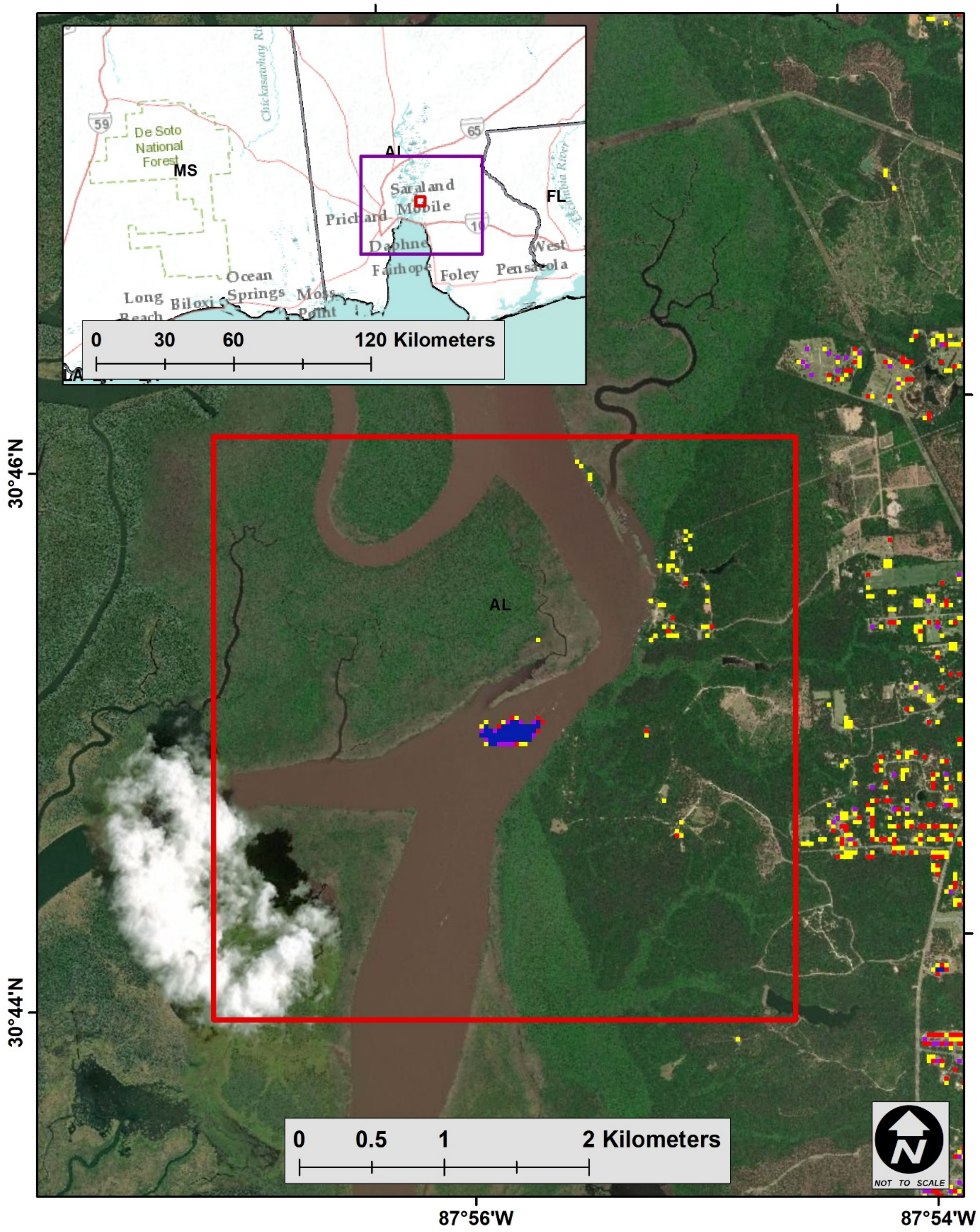

SI Figure 1 – ERR2 – Tensaw River in Alabama, showing building pixels within the river channel.

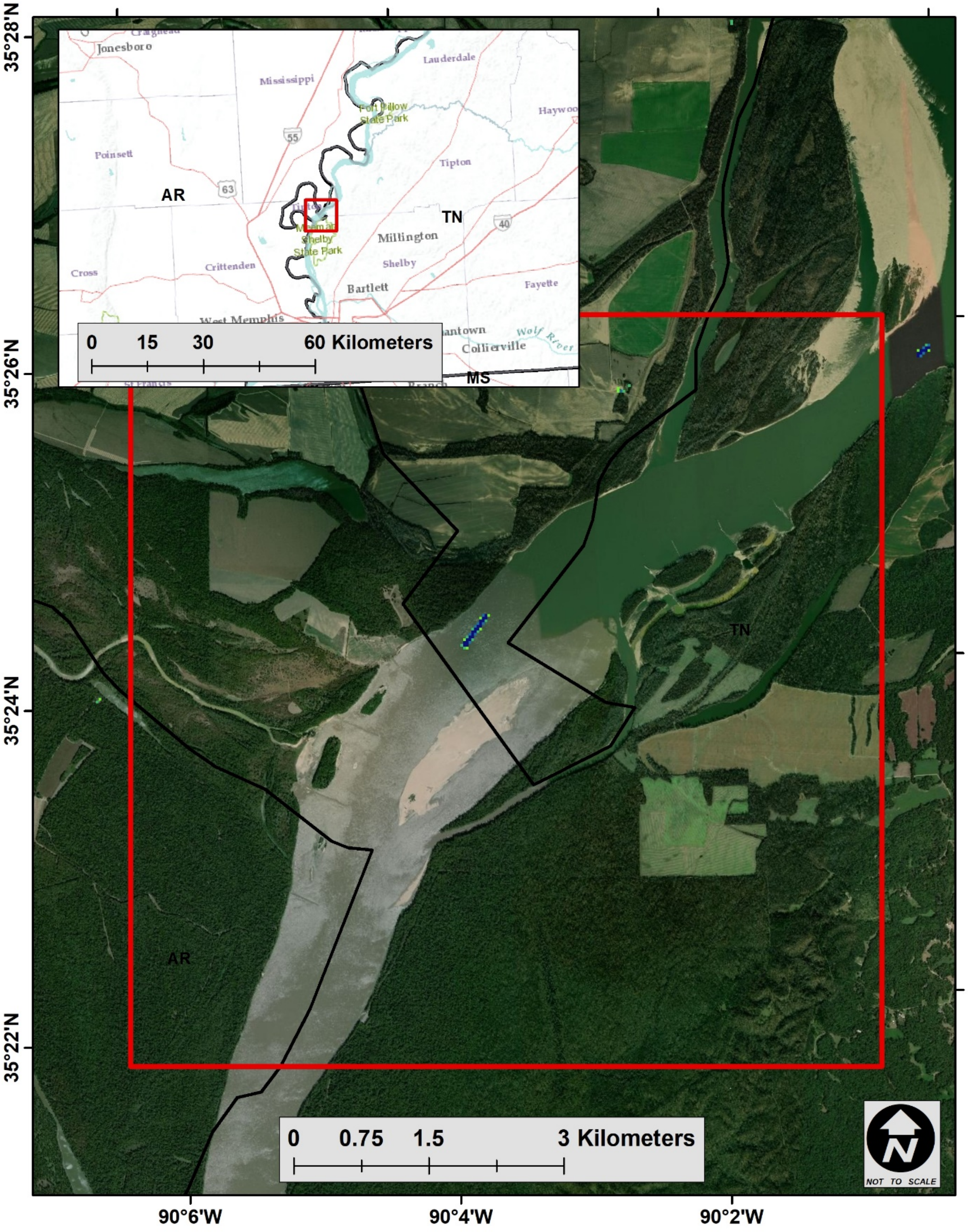

SI Figure 2 – ERR2 – Mississippi River in Arkansas, showing building pixels within the river channel.

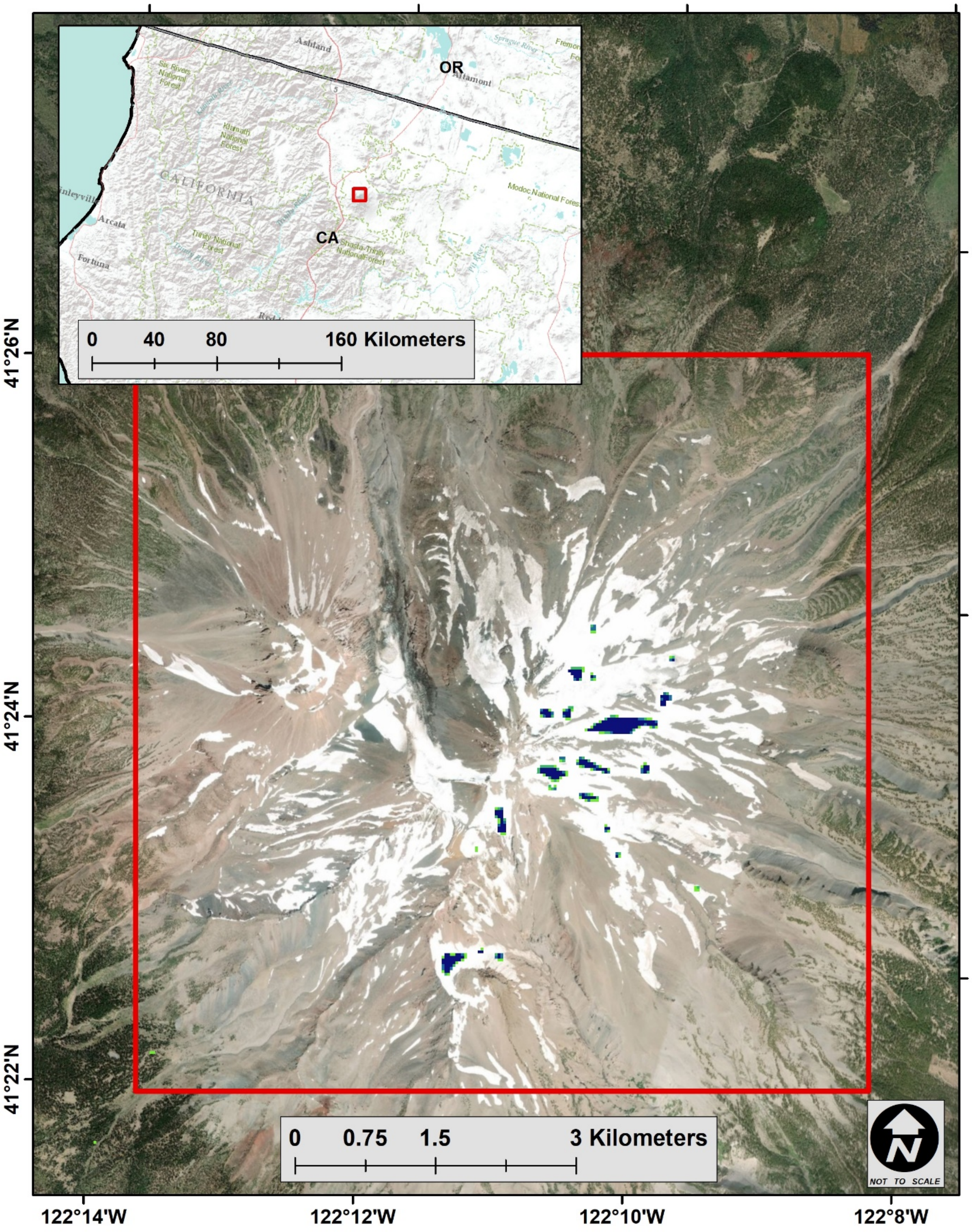

SI Figure 3 – ERR3 – Mt. Shasta, California, showing building pixels present within high-elevation glaciers.

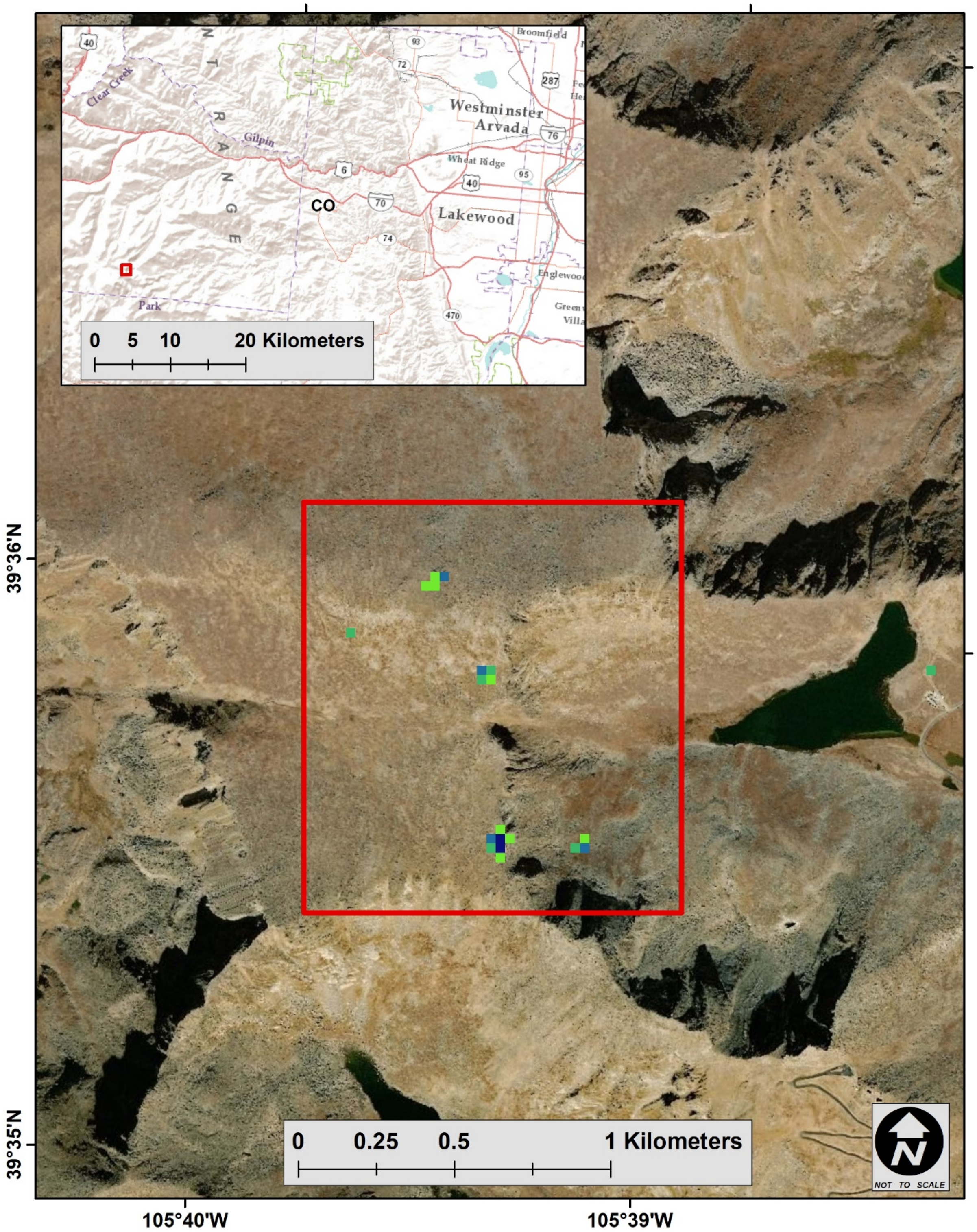

25

26 *SI Figure 4 – ERR3 – Mt. Evans, Colorado, showing building pixels present at high elevations.*

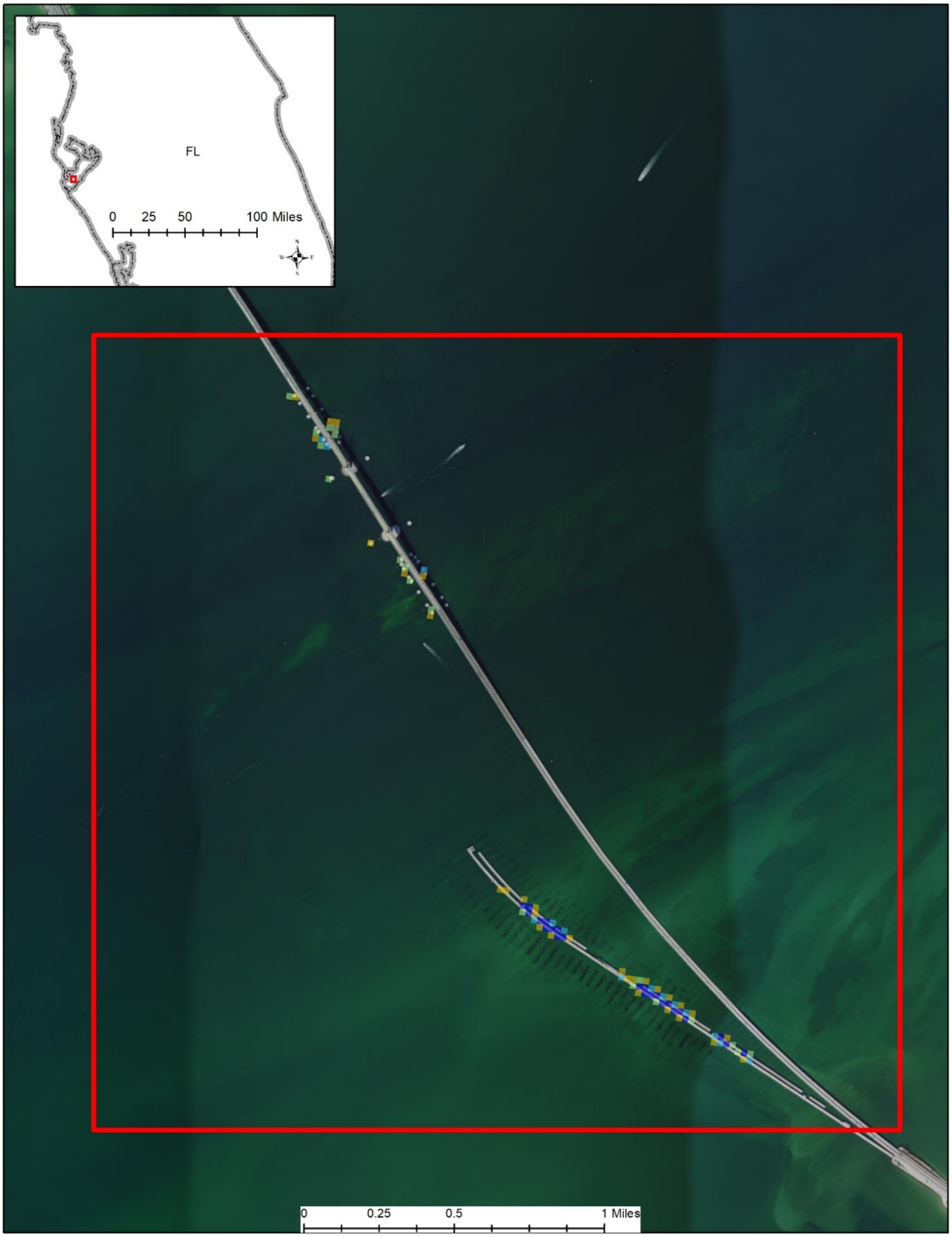

SI Figure 5 – ERR2; ERR7 – Sunshine Skyway Bridge, Tampa Bay, Florida, showing building pixels on the bridge and the pylons in water.

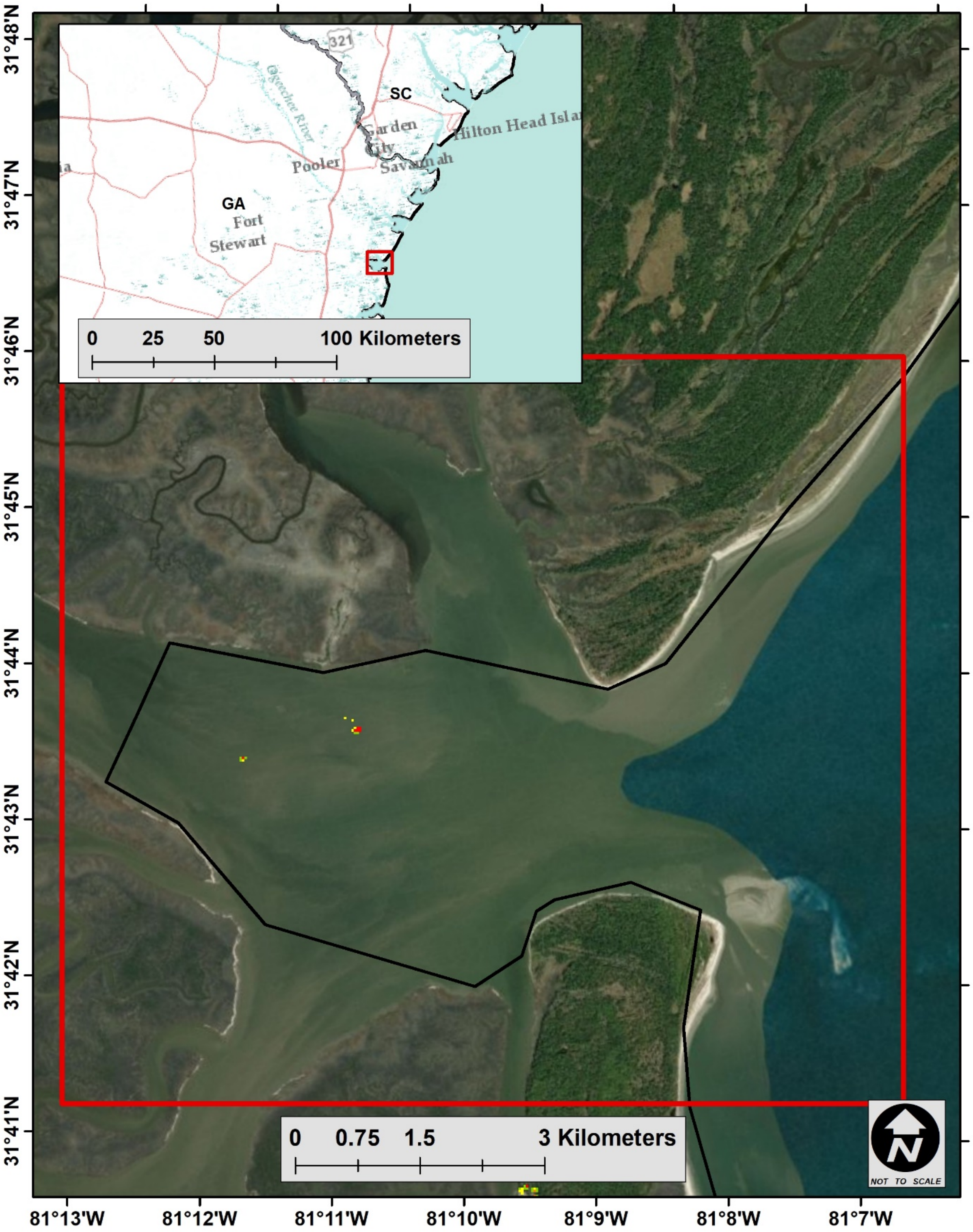

30

31 *SI Figure 6 – ERR2 – St. Catherine's Sound, Georgia, showing building pixels in open water.*

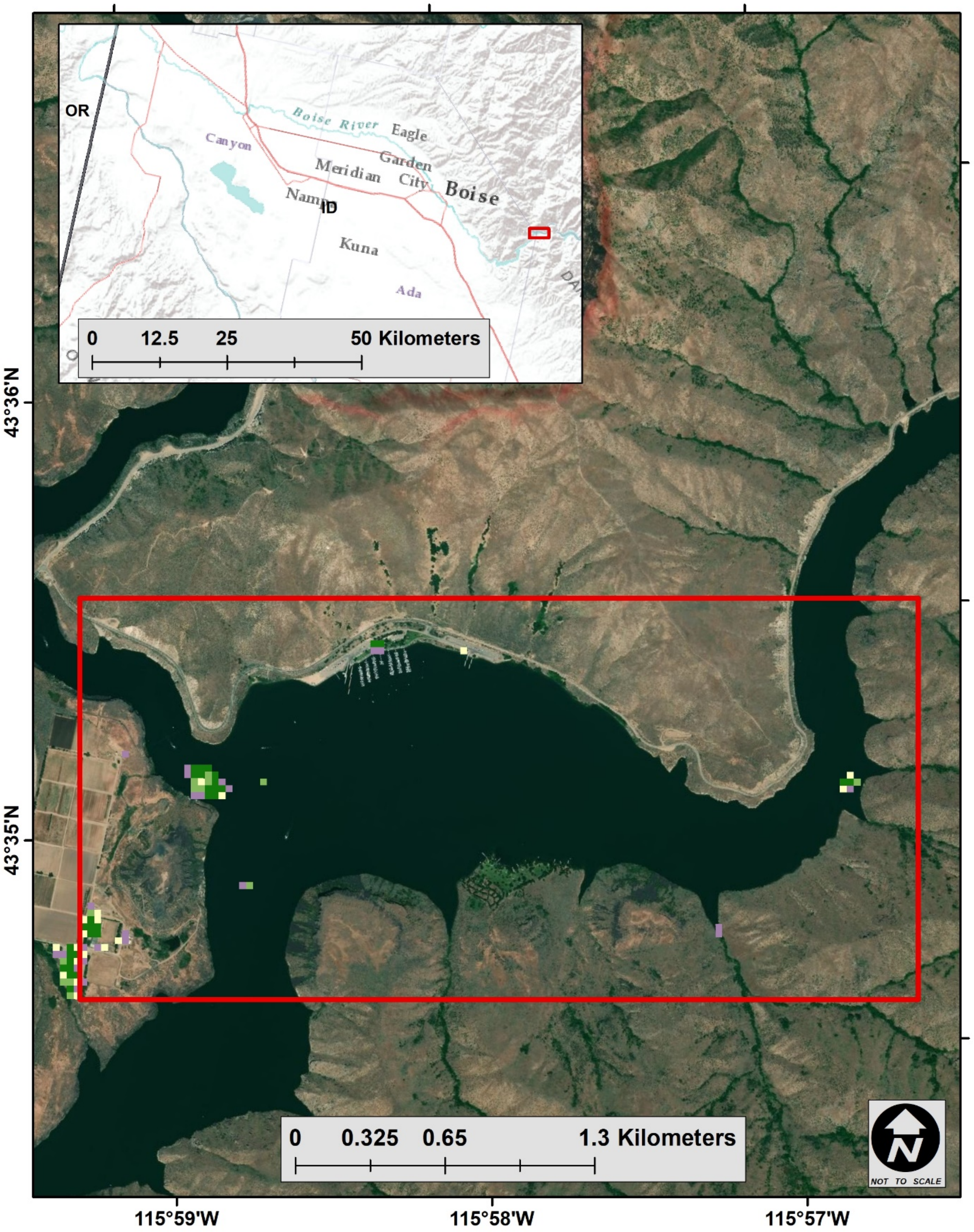

32

33 *SI Figure 7 – ERR2 – Lucky Peak Lake in Idaho, showing building pixels within the river channel.*

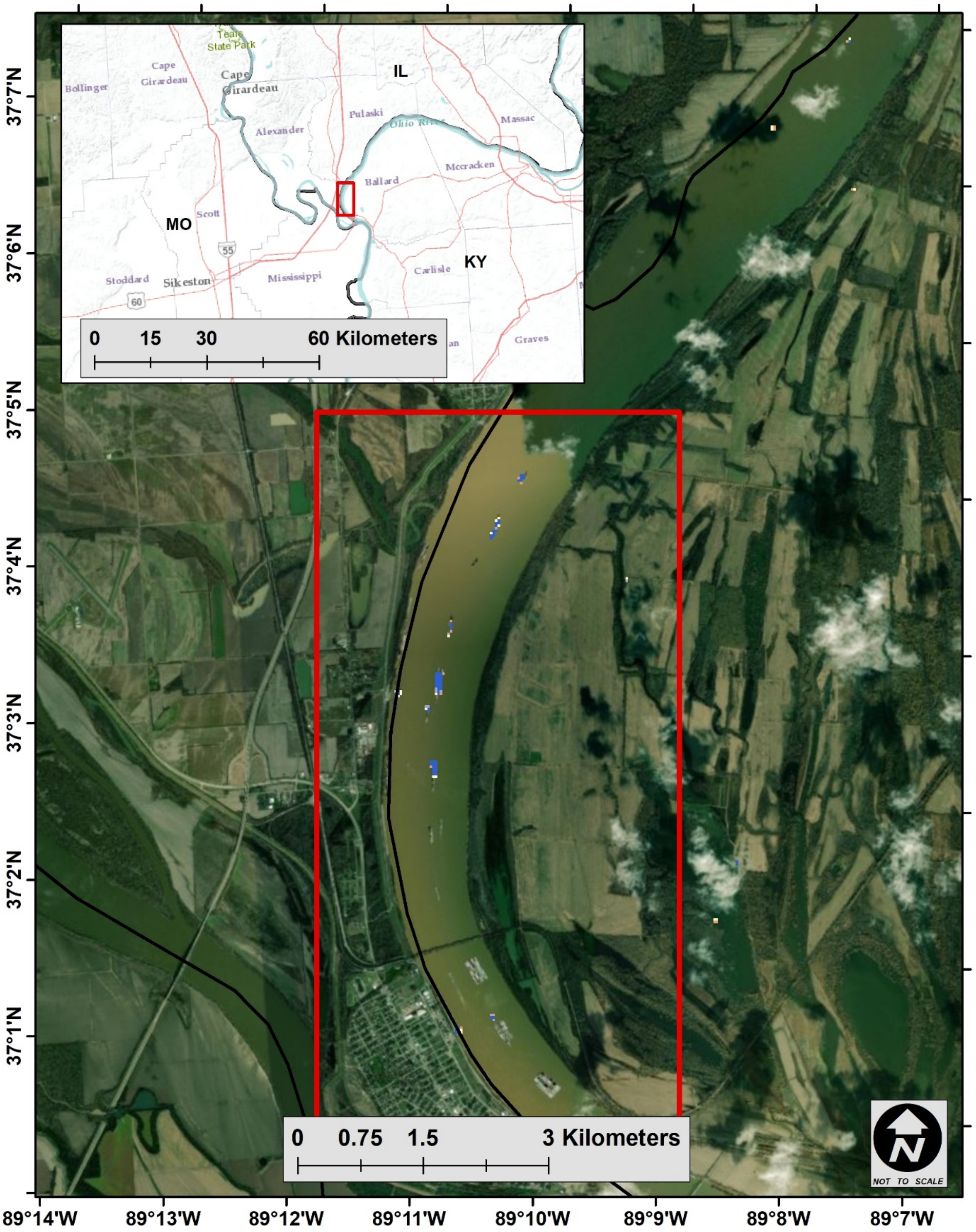

SI Figure 8 – ERR2 – Ohio River in Kentucky, showing building pixels within the river channel, some boats appear to be classified as buildings.

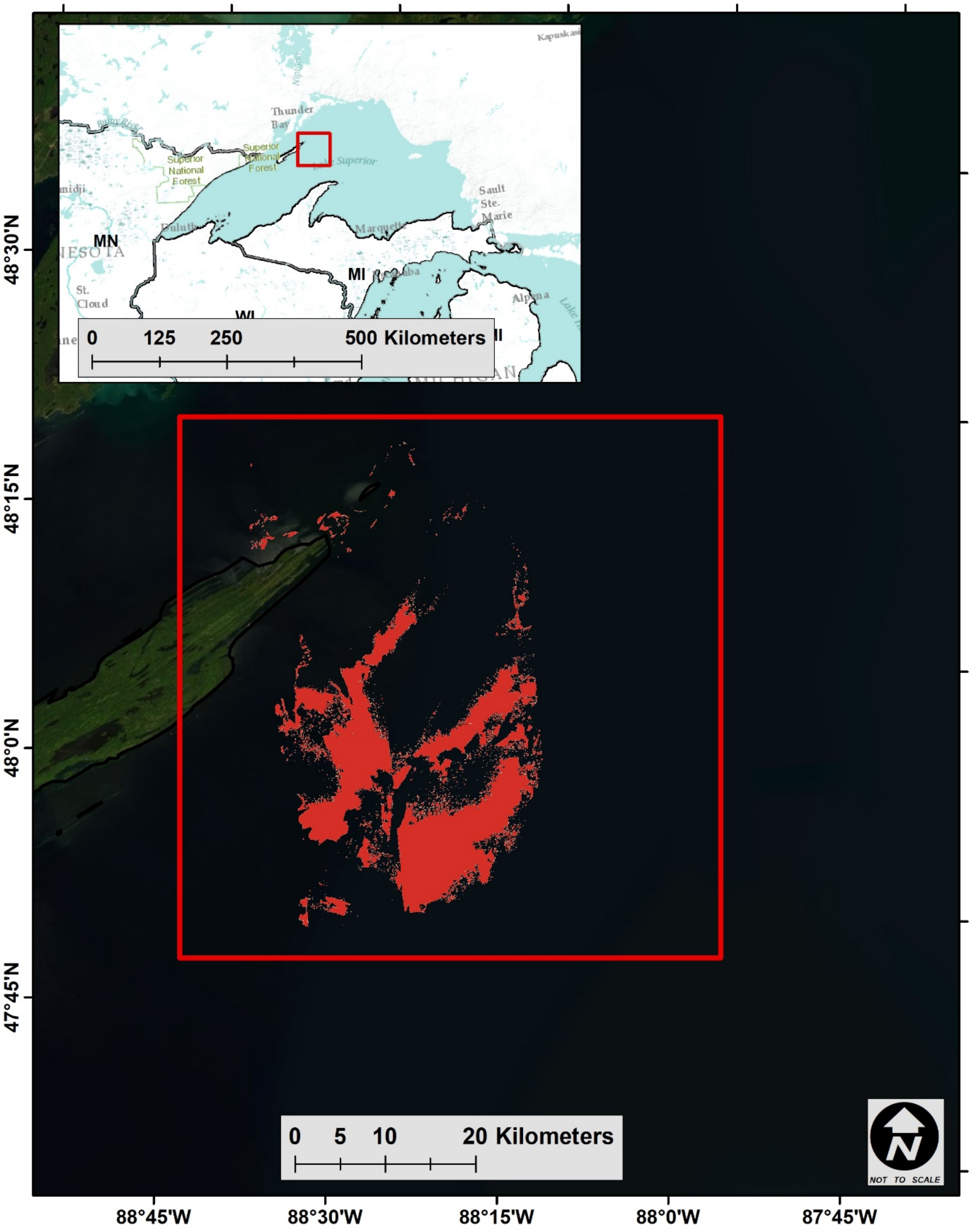

SI Figure 9 – ERR7 – Extent issue for Michigan. Areas are being classified as buildings in the middle of Lake Superior near Isle Royale National Park.

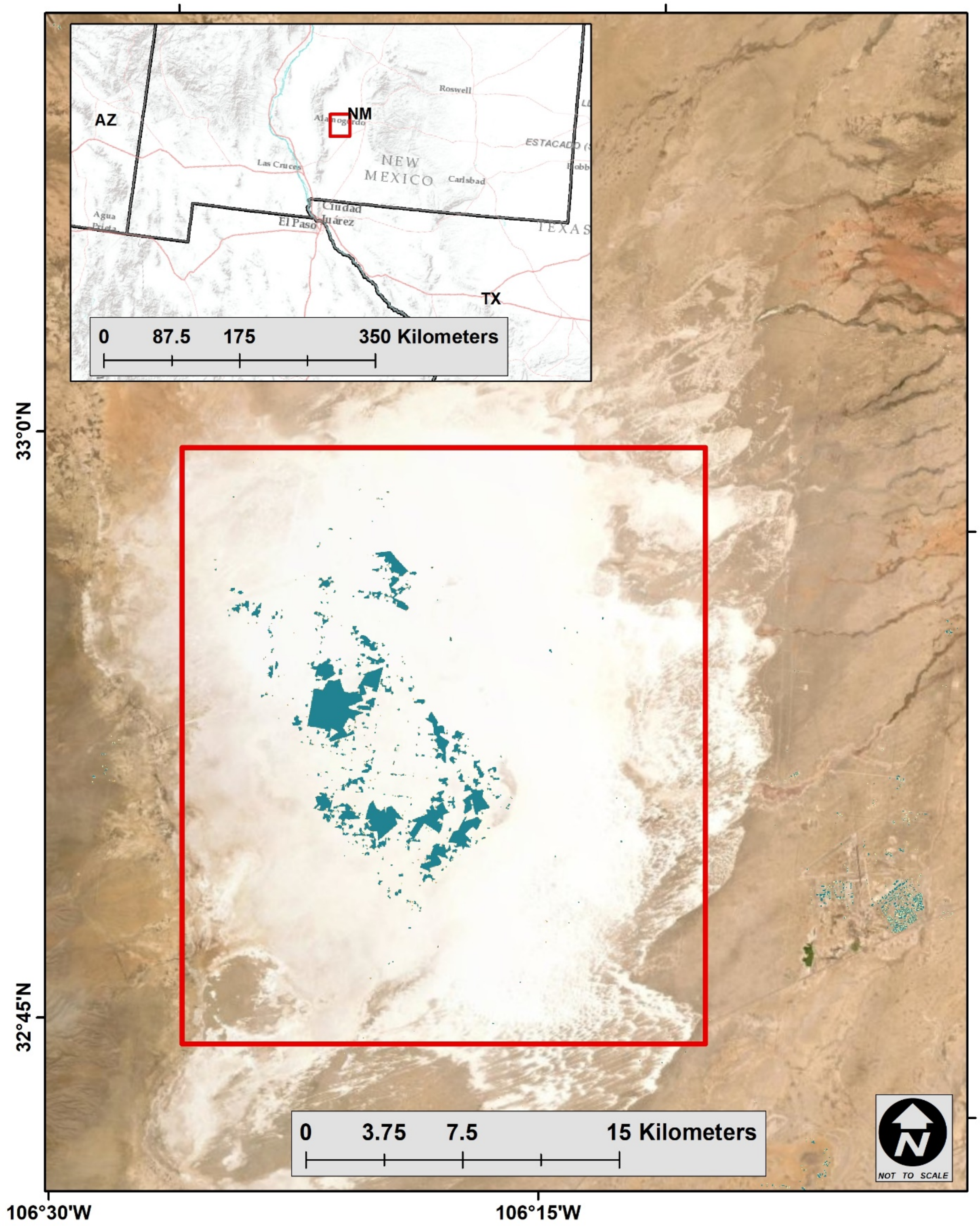

SI Figure 10 – ERR1; ERR4; ERR7 –New Mexico near White Sands National Park; reflectance issues are likely causing buildings to be generated on top of each other and in erroneous places.

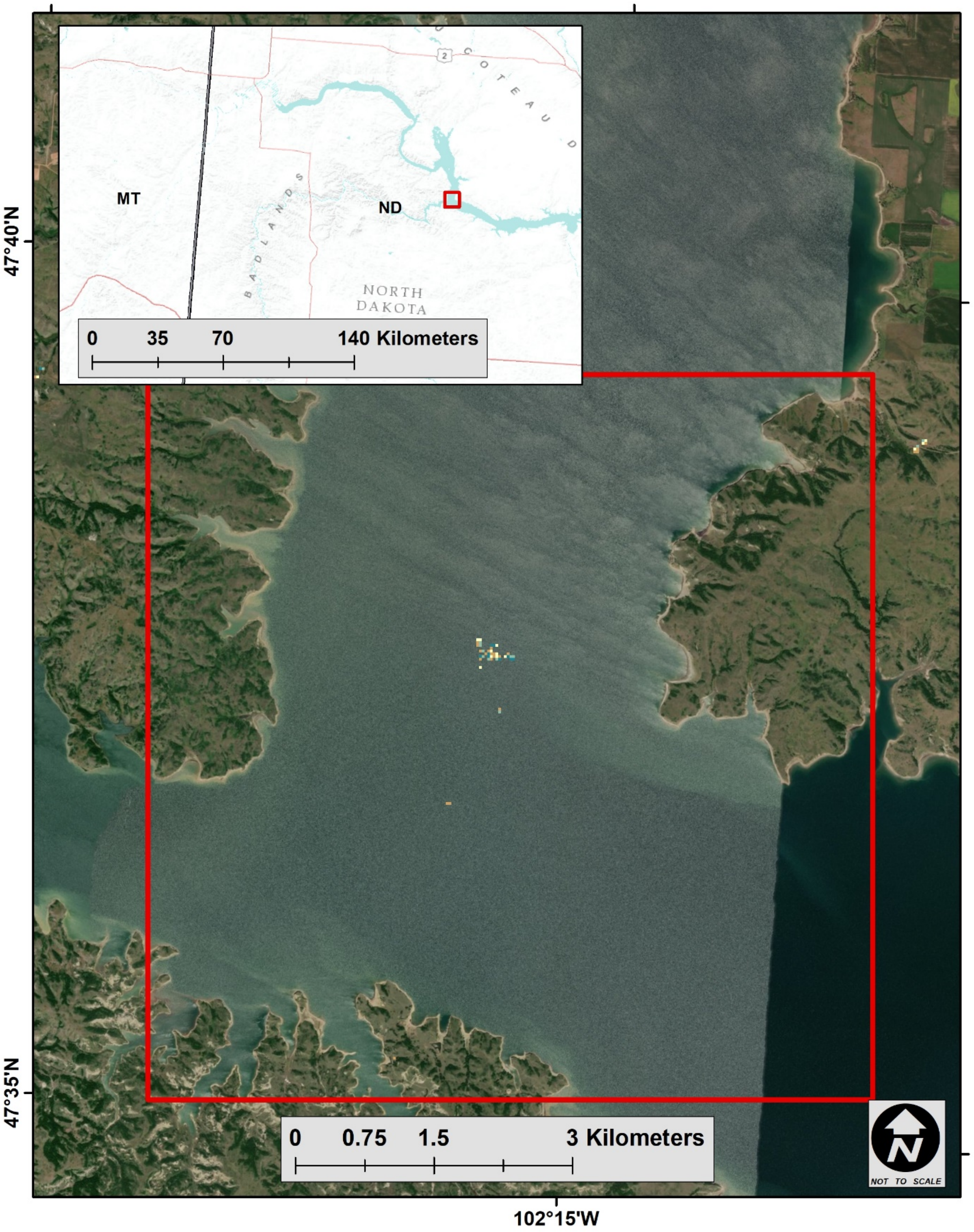

SI Figure 11 – ERR2 – Missouri River in North Dakota, showing building pixels within the river channel.

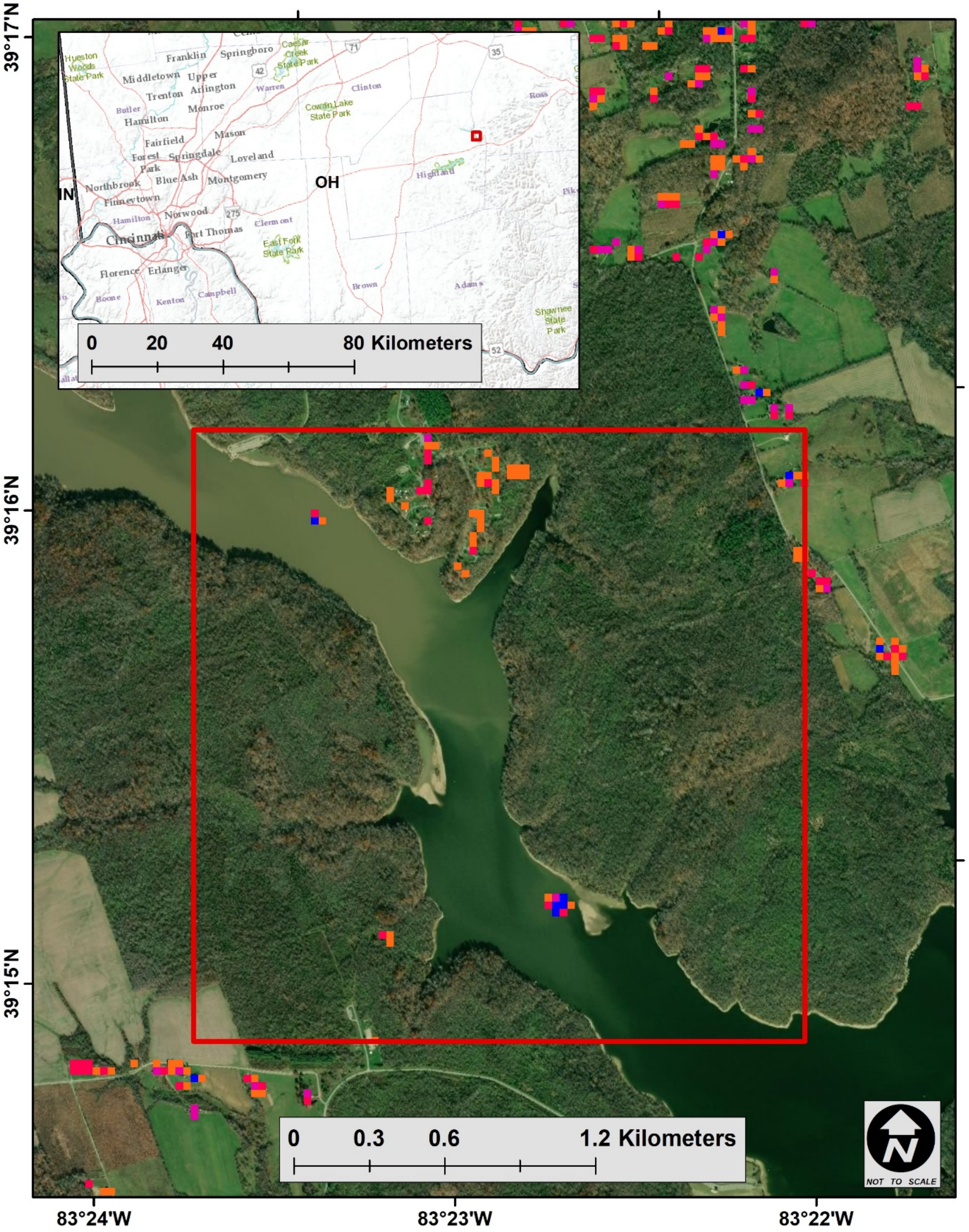

SI Figure 12 – ERR2 – Paint Creek Lake in Ohio, showing building pixels within the area of open water.

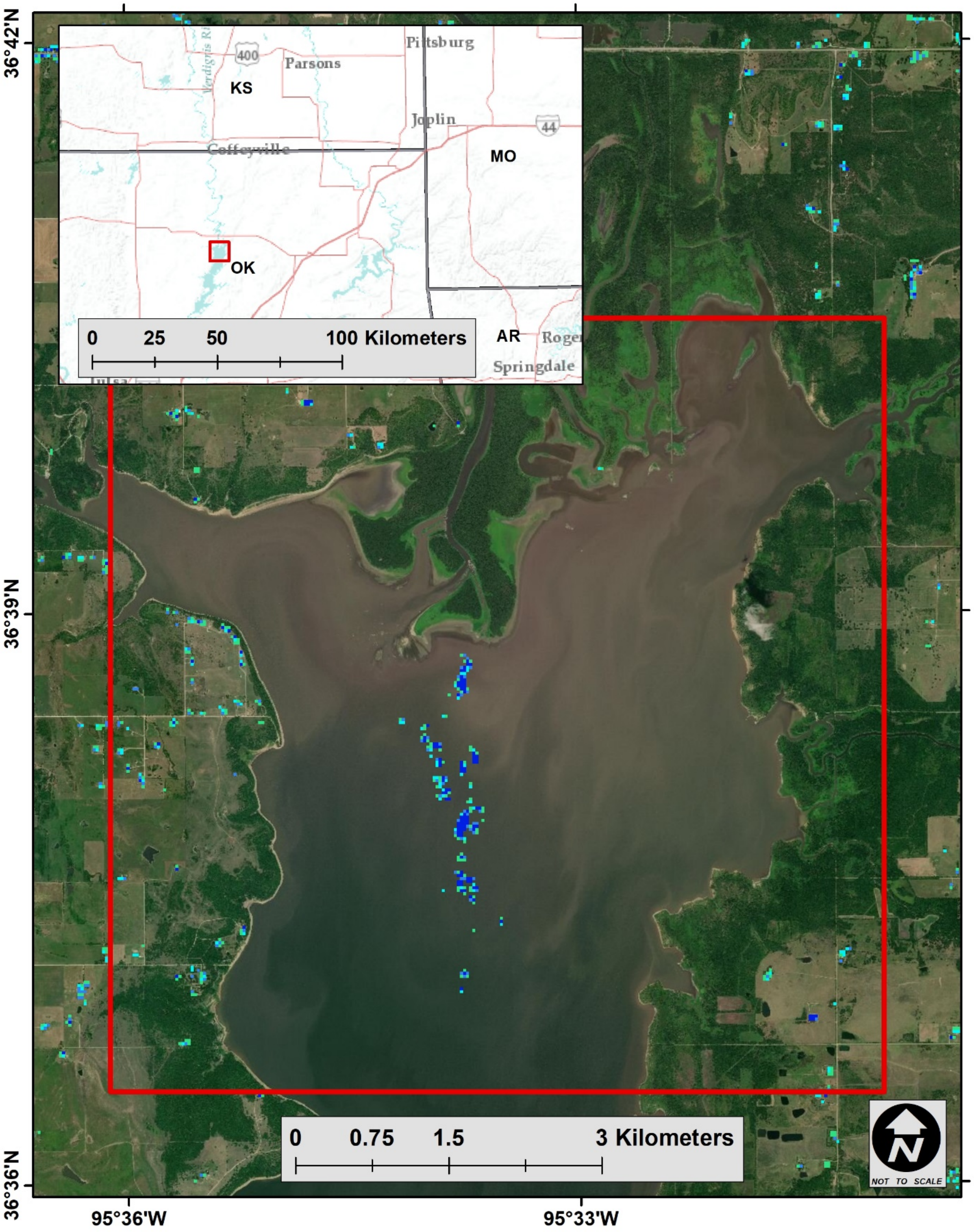

47

48 *SI Figure 13 – ERR2 – Oologah Lake in Oklahoma, showing building pixels within the area of open water.*

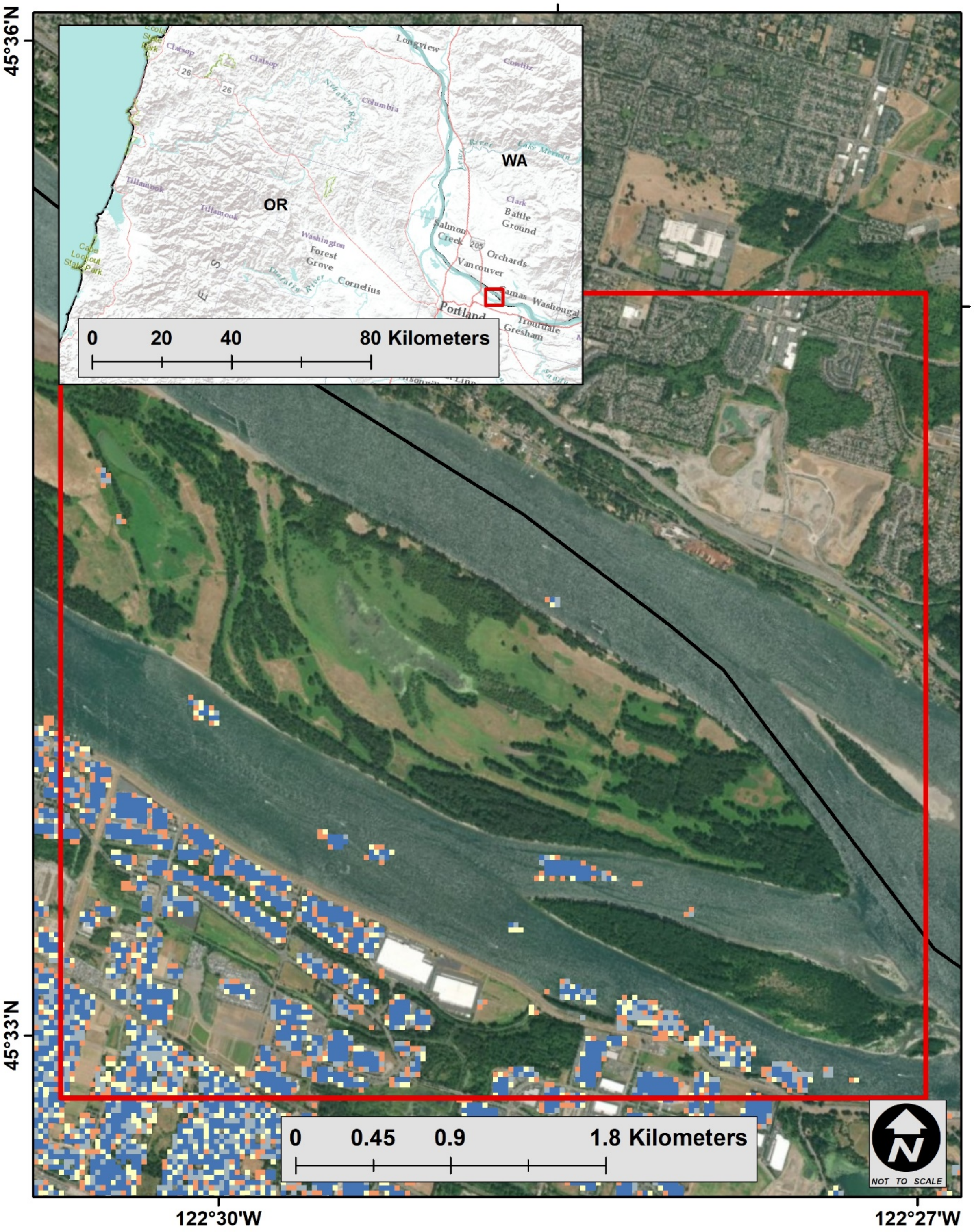

SI Figure 14 – ERR2 – Columbia River in Oregon, showing building pixels within the river channel.

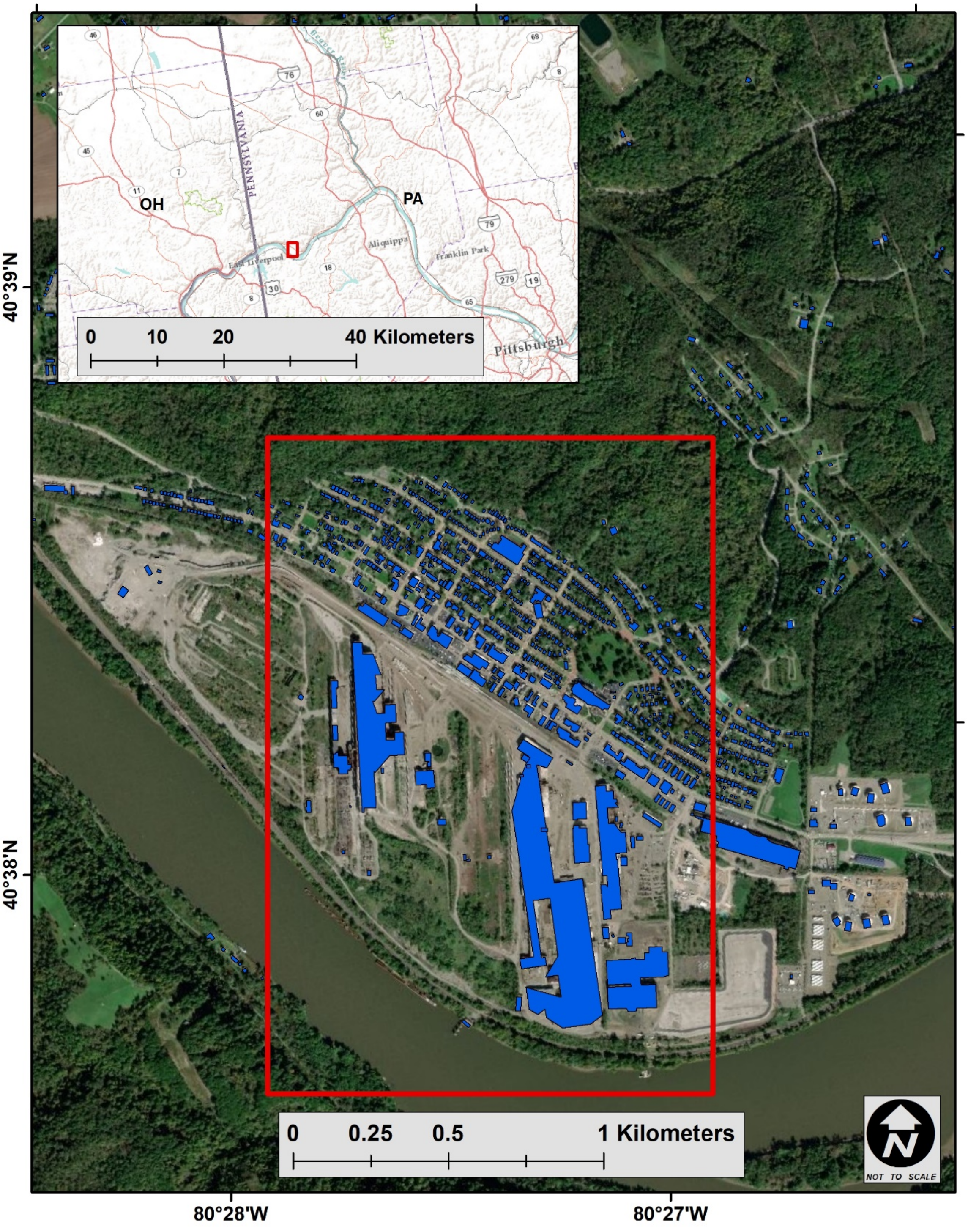

SI Figure 15 – ERR1; ERR2; ERR5 – Area in Midland, PA showing a railyard where trains are being classified as buildings causing overlapping polygons. There are also building polygons being generated in the river.

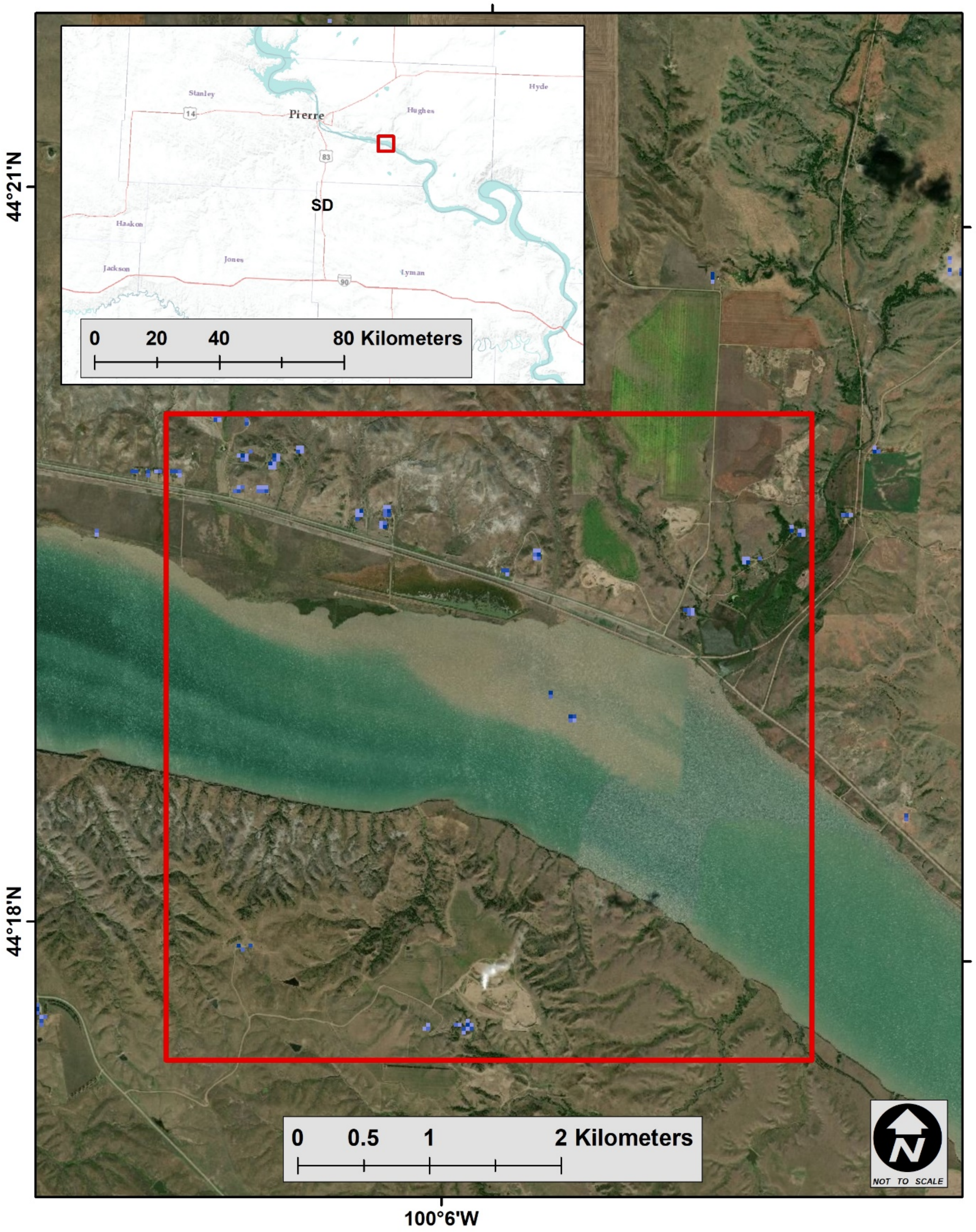

SI Figure 16 – ERR2 – Missouri River in South Dakota, showing building pixels within the river channel.

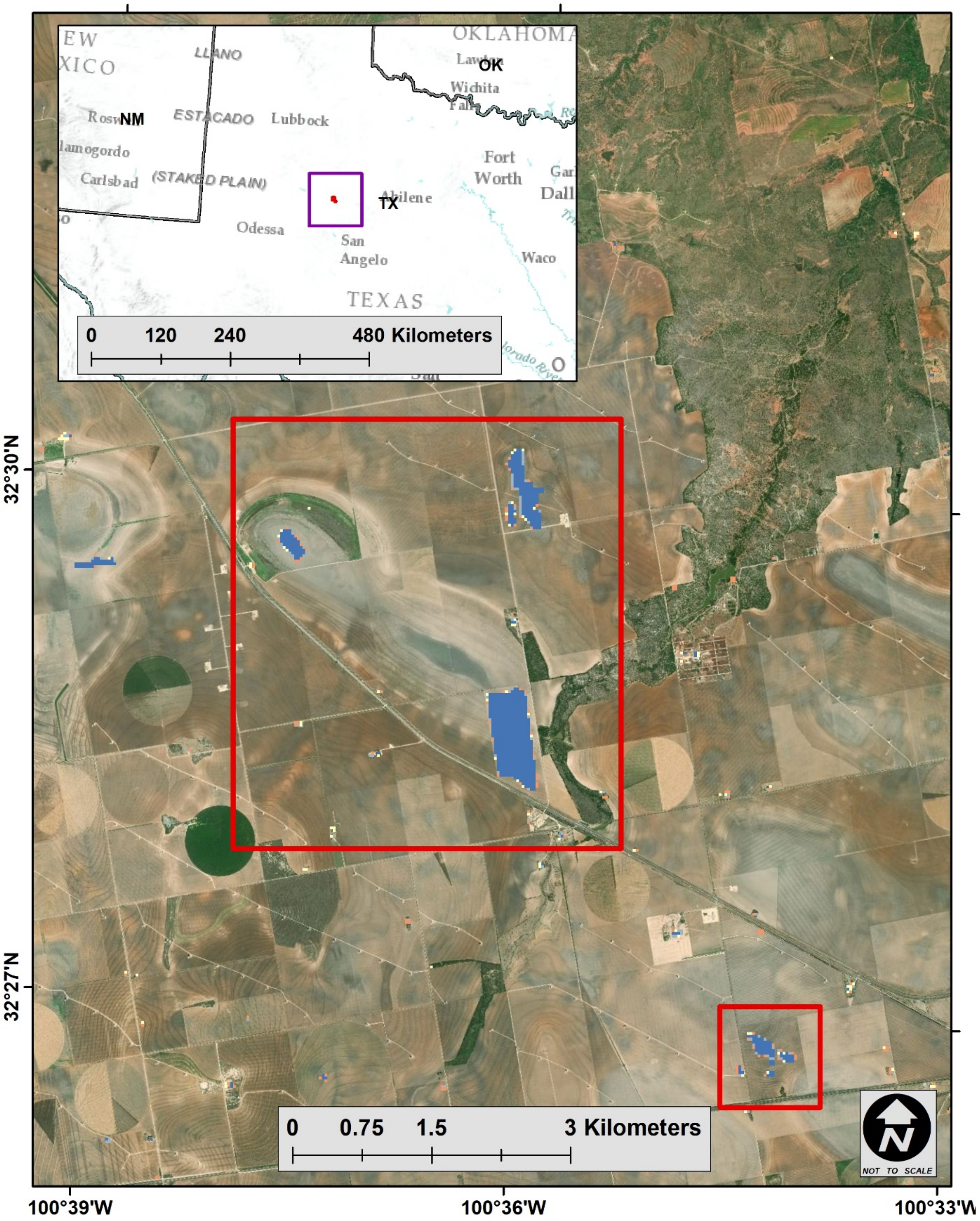

SI Figure 17 – ERR6 – Area near Sweetwater, TX, near wind turbines and mines that are showing large building footprints in bare ground areas.

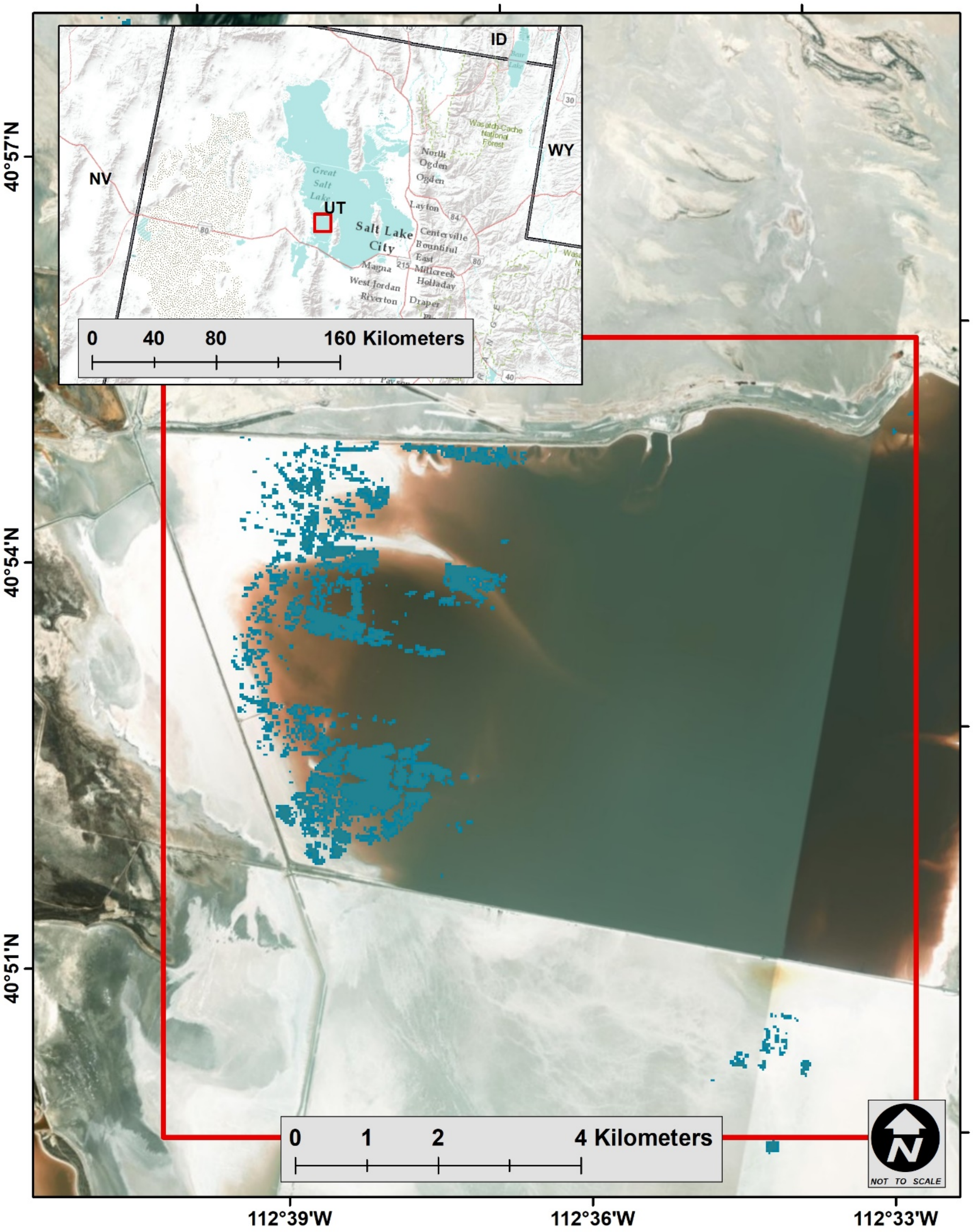

SI Figure 18 – ERR2; ERR4; ERR7 – Great Salt Lake, Utah, showing building pixels within open water boundary and salt deposits.

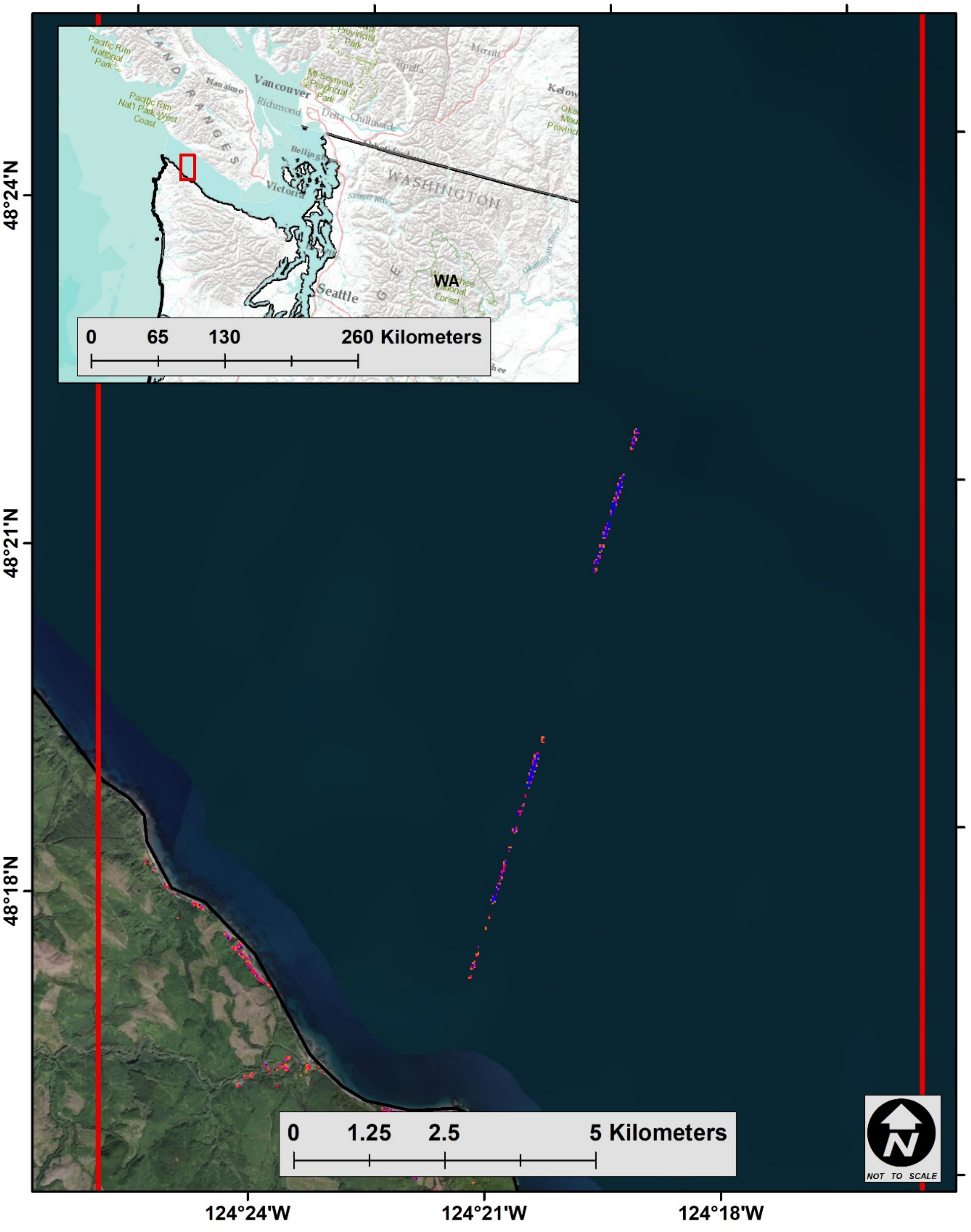

SI Figure 19 – ERR2 – Salish Sea, Washington, showing building pixels within the open water boundary. This can be caused by the contrast of color in the border to two different tiles that has created a linear pattern.
